# Computer aided designing of novel pyrrolopyridine derivatives as JAK1 inhibitors

**DOI:** 10.1038/s41598-021-02364-2

**Published:** 2021-11-29

**Authors:** Seketoulie Keretsu, Suparna Ghosh, Seung Joo Cho

**Affiliations:** 1 Department of Biomedical Sciences, College of Medicine, Chosun University, 375 Seosuk-dong, Dong-gu, Gwangju, 501-759 Republic of Korea; 2 Department of Cellular Molecular Medicine, College of Medicine, Chosun University, Gwangju, 501-759 Republic of Korea

**Keywords:** Computational biology and bioinformatics, Computational models

## Abstract

Janus kinases (JAKs) are a family of non-receptor kinases that play a key role in cytokine signaling and their aberrant activities are associated with the pathogenesis of various immune diseases. The JAK1 isoform plays an essential role in the types 1 and II interferon signaling and elicits signals from the interleukin-2, interleukin-4, gp130, and class 2 receptor families. It is ubiquitously expressed in humans and its overexpression has been linked with autoimmune diseases such as myeloproliferative neoplasm. Although JAK1 inhibitors such as Tofacitinib have been approved for medical use, the low potency and off-target effects of these inhibitors have limited their use and calls for the development of novel JAK1 inhibitors. In this study, we used computational methods on a series of pyrrolopyridine derivatives to design new JAK1 inhibitors. Molecular docking and molecular dynamics simulation methods were used to study the protein-inhibitor interactions. 3D-quantitative structure–activity relationship models were developed and were used to predict the activity of newly designed compounds. Free energy calculation methods were used to study the binding affinity of the inhibitors with JAK1. Of the designed compounds, seventeen of the compounds showed a higher binding energy value than the most active compound in the dataset and at least six of the compounds showed higher binding energy value than the pan JAK inhibitor Tofacitinib. The findings made in this study could be utilized for the further development of JAK1 inhibitors.

## Introduction

Janus kinases (JAK) are non-receptor tyrosine kinases that mediate cellular signaling from the extracellular matrix via the cytokine receptors^1^. They play a crucial role in type 1 and type 2 cytokines mediated signaling^2^. The binding of endogenous ligands such as cytokines and interferons at the transmembrane receptors leads to the activation of JAKs, which in turn phosphorylate the transcription factor STAT kinases^3^. The activated STAT kinases modulate the expression of genes that are associated with cell proliferation, apoptosis, and angiogenesis. The dysregulation of the JAK/STAT signaling has been linked with various autoimmune disorders. The JAK family consists of the isoforms JAK1, JAK2, JAK3, and the tyrosine kinase 2 (TYK2)^4^. While the JAK1 and JAK2 are ubiquitously expressed, the TYK2 is predominantly expressed in the endothelial. The JAK1 isoform regulates cytokine signaling from the interleukin-2 (IL2) receptor, interleukin-4 (IL4) receptor, and glycoprotein 130 receptor families. They also transduce signaling from type 1 and type 2 interferons^5^. The aberrant activity of JAK1 is linked with the pathogenesis of inflammatory disorders and immune diseases like rheumatoid arthritis (RA), ulcerative colitis, and Crohn’s disease^6,7^.

The JAK1 shares a high sequence identity with the other members of the JAK family. Structurally, JAK1 consists of seven distinct domains termed JAK homology (JH1-JH7) domains. The JH1 domain consists of the protein tyrosine kinase domain and plays a significant role in physiological function. The JH2 domain consists of a kinase-like domain called the pseudokinase domain that regulates the activity of the protein tyrosine kinases (PTK) domain. The JH3 and JH4 share some homology with the Src homology 2 (SH-2) domains and stabilize the conformation of JAK1. The JH5 and JH7 domains make up the N-terminus of JAK1 and play an essential role in the regulation of the JAK1. The ATP binding takes place at the cavity formed by the residues from the glycine-rich loop, activation loop, hinge region, and αC-helix at the JH2 domain.

Currently, JAK1 inhibitors such as Filgotinib, Tofacitinib, Peficitinib, and several other second-generation inhibitors are in consideration for the treatment of inflammatory disorders and autoimmune disorders^8–10^. Tofacitinib was approved for oral use against RA. However, it is a multi-JAK inhibitor with inhibitory activity against JAK2, JAK3, and TYK2^11^. Filgotinib is a second-generation, orally available drug in clinical trials for RA. However, the drug is a potent inhibitor of JAK1, JAK2, and TYK2^12^. Peficitinib is another pan JAK inhibitor that has been approved for clinical use in Japan. Clinical trials have shown that Peficitinib is effective against RA with an acceptable safety profile^10,13^. Several other JAK1 inhibitors are also in consideration for the treatment of autoimmune and inflammatory diseases^14^. However, due to the low potency, non-selectivity, and off-target effects of existing drugs, developing novel JAK1 inhibitors with high potency is in crucial need for the treatment of RA.

We used computational modeling techniques such as 3D-QSAR, molecular docking, molecular dynamics simulation, and free energy calculation methods to study a series of pyrrolopyrimidine based JAK1 inhibitors. The study was carried out to explore the protein–ligand interactions and to characterize structural features important for the inhibition of JAK1. 3D-QSAR models were developed for structure–activity relationship analysis and to predict the activity value of novel JAK1 inhibitors. The relative binding affinity of the inhibitors and the newly designed compounds were calculated using free energy calculation methods.

## Methodology

### Data collection and protein preparation

The dataset of 50 pyrrolopyrimidine based compounds that showed inhibitory activity for JAK1 was collected from the literature^15^. The half-maximal inhibitory concentration (IC_50_) values were converted to negative log values (pIC_50_). The inhibitory activity (pIC_50_) values of the compounds ranged from 5 to 9.7. The structure of the dataset compounds and the activity values are given in Table 1. The X-ray crystallographic structure of JAK1 in complex with the Tofacitinib (PDB ID **3EYG**) at 1.9 Å resolutions was collected from the protein repository^16^. The protein structure was prepared by removing solvents molecules and adding the missing atoms.

**Table 1:**
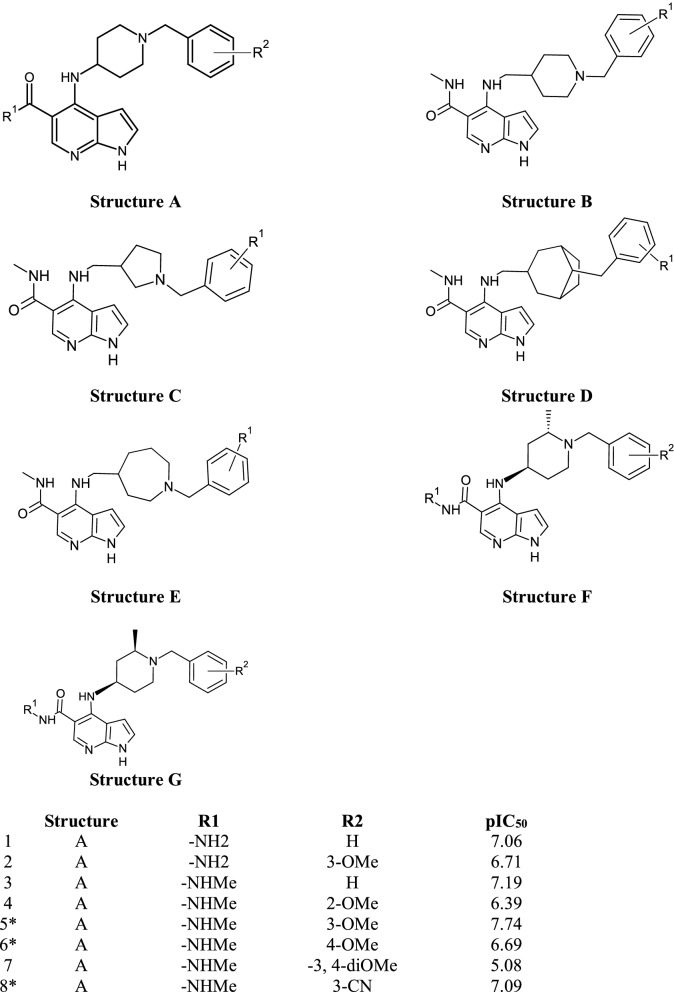

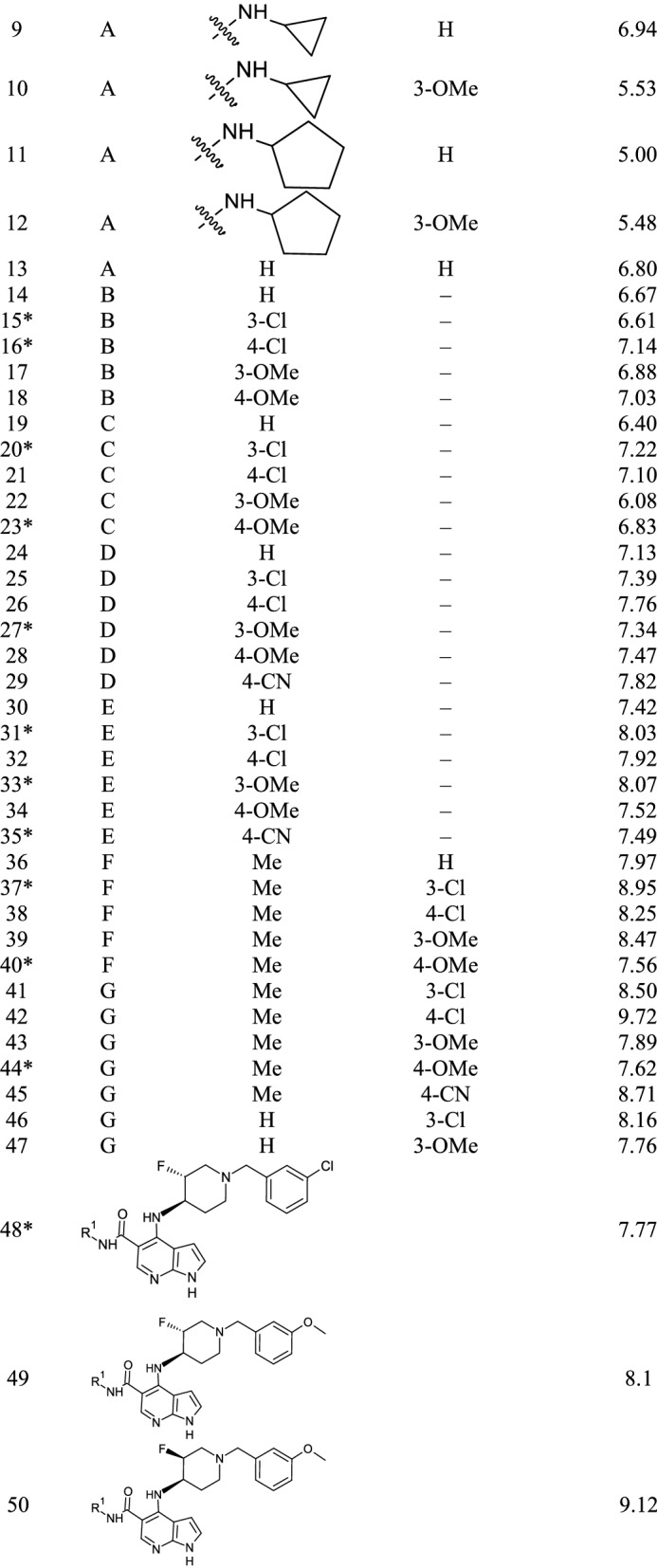
Dataset of pyrrolopyridine based JAK1 inhibitors.

*Test set compounds.

### Molecular docking

The protein–ligand binding was carried out using Autodock 4.2 (The Scripps Research Institute, La Jolla, CA, USA)^17^. Prior to the docking, the protein was prepared by adding polar hydrogen atoms, followed by the addition of Kollman charges. The inhibitor was sketched and minimized in Sybyl X 2.1 (Tripos Inc., St. Louis, MO, USA). Gasteiger charges were added as partial charges. The conformational search area within the receptor was defined by a 60 × 60 × 60 grid box. The gird space was set to 0.37 Å. The Lamarckian Genetic algorithm within the Autodock program was used to perform the conformational search and generate 100 conformations. The autodock tools package was used to analyze the outcome of the docking^17^. The protocol described above was used during the docking of the dataset compounds as well as the docking of the newly designed compounds.

### Molecular dynamics

Molecular dynamics simulation was performed using the Gromacs 2020^18^. The docked protein–ligand structure from the docking study was used as the initial structure for simulation. The ACPYPE (or AnteChamber PYthon Parser interfacE) script was used to develop the ligand parameters^19,20^. The amber force field Amber99SB was used to parameterize the protein^20^. The protein–ligand complex was placed inside a cubic box and was solvated with a TIP3P water model. Na^+^ ions and Cl^−^ ions were added to the system to neutralize the system. The system was energy minimized with the maximum force F_max_ set to 1000 kJ mol^−1^ nm^−1^ to remove steric contacts. Position restrained NVT ensemble and NPT ensemble equilibrations were carried out to gradually heat the system to a pressure and temperature of 1 bar and 300 K, respectively. During the equilibration, V-rescale was used for temperature coupling and Parrinello-Rahman was used for pressure coupling. The long-range electrostatic interactions were was estimated using the Particle Mesh Ewald method^20^. During the simulation, hydrogen bonds were constrained using LINCS methods and the minimum time step was kept at 2 fs. The unrestrained production was performed using the leap-frog integrator. A more detailed explanation of the simulation methodology can be found in previous protein–ligand interaction studies by our group^21–24^. Protein–ligand snapshots were collected from the equilibrated region of the MD simulation and the binding energy values were estimated using the Molecular mechanics Poisson–Boltzmann surface area (MM-PBSA) method implemented by Kumari et al.^25^.

The *g-mmpbsa* method implemented by Kumari et al.^25^ does not consider the entropy term hence, the methods can only give the relative BE. Though there are several methods to calculate the entropy term^26,27^, these methods are time-consuming and the magnitude of the standard error is high. Hence, the binding energy results presented in the study are considered as relative binding energy values. More detail regarding the MM/PBSA methods can be found in the supplementary material.

### Three dimensional structure–activity relationships (3D-QSAR)

The compounds were sketched based on the binding conformation of the most active compound 42 inside JAK1 as observed in the MD simulation. The compounds were prepared by adding Gasteiger charges as partial charges and then minimizing the substituents in Sybyl X 2.1 (Tripos Inc., St. Louis, MO, USA). Using the distill-rigid alignment method, the compounds were superimposed on the common substructure, using the most active compound as the template.

3D-QSAR models were developed to analyze the relationship between the physicochemical descriptor of the compounds and their biological activity values (pIC_50_). The dataset was split into test sets and training set for training and validating the models using a stratified random sampling approach. As a first step, the compounds were split into various mutually exclusive and non-overlapping groups (or strata) based on the pIC_50_ values. Based on a random draw, compounds belonging to each group were split into a training set and test set in a 2:1 ratio. The final training set consists of the training sets compounds from all the groups. Similarly, the final test set consists of the test set compounds from all the groups. This process of drawing test set and training set was repeated five times resulting in five sets of the test set and training set pairs (Sets 1–5).

Taking the first test set and training set pair (Set 1), Comparative Molecular Field Analysis (CoMFA) models were developed for various charge schemes namely Gasteiger, Gasteiger-Hückel, Delre, Pullman, and MMFF94^28,29^. In CoMFA, the electrostatic and steric descriptors were used as the independent variables and the activity values of the compounds were used as the dependent variables. The models were validated using various internal and external validation techniques. The non-validated Leave-One-Out (LOO) method was used to estimate the predictive ability of the models.

The bootstrapping method was used to evaluate the robustness of the model. The sensitivity of the model to chance correlation was tested using the progressive scrambling method (progressive scrambling Q^2^). To evaluate the predictive ability of the models against the external dataset, the $r_{pred}^{2}$, $Q_{F1}^{2}$*,* $Q_{F2}^{2}$*,*$Q_{F3}^{2}$*,* Concordance Correlation Coefficient (CCC), mean absolute error (MAE), and $r_{m}^{2}$ validations were performed^30^. A more detailed description of the validation techniques is provided in the Supplementary Material. Comparative Molecular Similarity Index Analysis (CoMSIA) models were also developed based on various combinations of electrostatic, steric, H-bond donor, and acceptor and hydrophobic descriptors, and the models were tested using the various validation techniques. Finally, based on statistical results, the models with the highest *q*^2^, *r*^2^, and $r_{pred}^{2}$ values were selected for further contour map study. We have also analyzed the applicability domain (AD) of the 3D-QSAR models to assess their applicability and reliability in predicting the activity values of the test set compounds. The williams plots showing the standard residuals and the leverage are provided below. In William’s plot, the standard residuals value within the range of + 3 and − 3 are considered acceptable and the leverage value less than h* = 3(*p* + 1)/*n* where, *p* is the number of descriptors and *n* is the number of compounds.

## Results

### Molecular docking

The binding of the most active compound 42 with JAK1 was carried out to study the protein–ligand interaction. The Lamarckian Genetic algorithm in Autodock 4.2 was used to generate a maximum of 100 ligand conformations. Analysis of the result was carried out by clustering the conformations of the ligand-based on root mean square deviation (RMSD) and a representative structure was selected based on nonbonded interactions and binding affinity score. The docking procedure was validated by docking the ligand (Tofacitinib) which was reported in a complex with JAK1 (PDB **3EYG**) from an X-ray crystallography experiment^16^. The docked pose of Tofacitinib overlapped closely with that of the crystalized structure. The overlap between the docked pose and crystal pose of Tofacitinib is shown in Fig. S1a (Supplementary Material). The compound 42 was bound to JAK1 with a binding affinity score of − 10.2 kcal/mol. The interactions between compound 42 and JAK1 are shown in Fig. S1b (Supplementary Materials). The pyrrolopyridine moiety of the compound formed H-bond interactions with F958 and L959 at the hinge between the N-lobe and the C-lobe domains. The methyl group of the methyl piperidine moiety extended out of the binding pocket and was exposed to the bulk solvent. The chlorobenzyl moiety folded inward into the hydrophobic pocket formed by the residues from the activation loop, αC-helix, and the P-loop. This folding of the chlorobenzyl moiety towards the hydrophobic pocket was reminiscent of the conformation observed in the crystallographic structure of the selective JAK1 inhibitor LKT (PDB ID **6SM8**) reported by Su et al.^31^. The binding conformation of compound 42 selected from the docking analysis was prepared for further molecular dynamics simulation study.

### MD simulation and binding energy (BE) calculation

**Figure 1:**
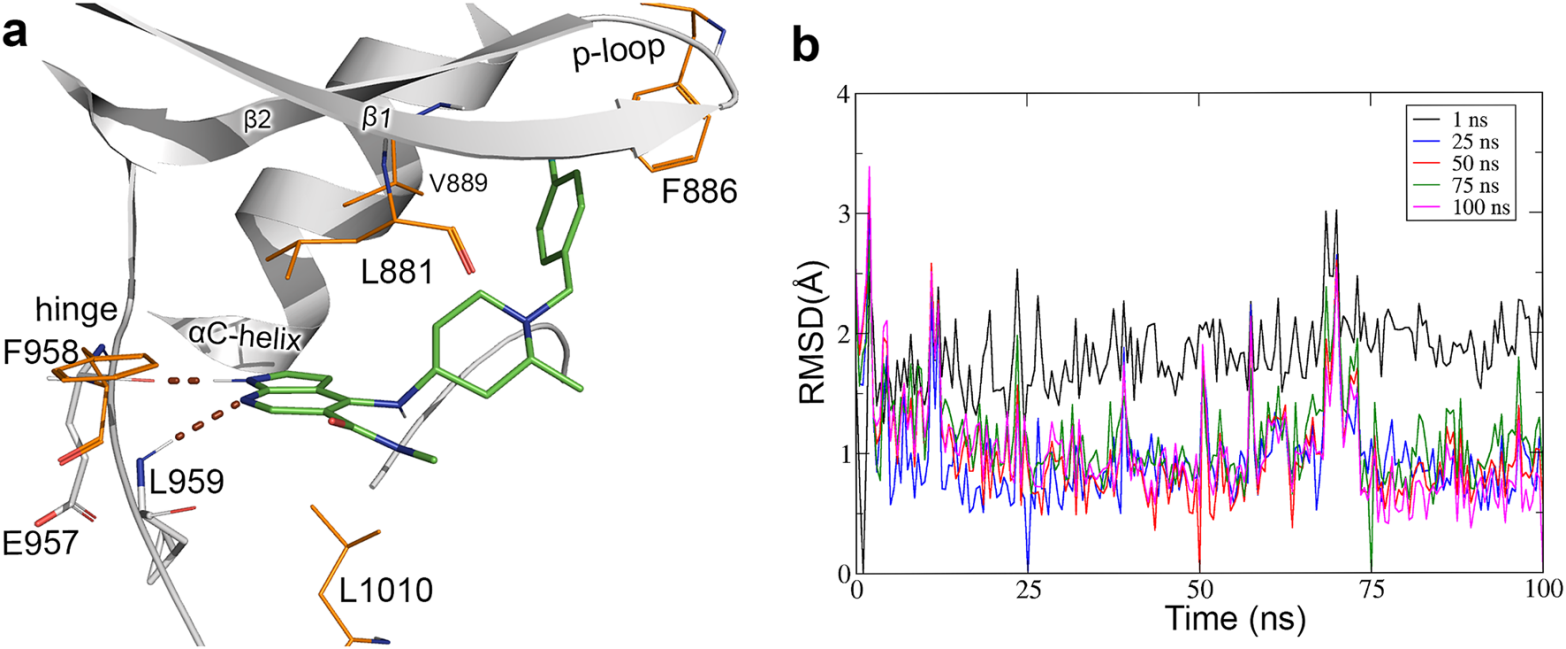
H-bond interactions of the most active compound (**42**) and JAK1 from the MD simulation. (**a**) Binding interactions between compound 42 (green) and JAK1 (grey). H-bond interactions are represented by brown dotted lines and residues forming H-bond interactions are shown in grey stick representations. Residues that formed hydrophobic interactions are shown in orange color lines. (**b**) Root mean square deviation (RMSD) of the ligand (compound 42) with respect to various snapshots (1 ns, 25 ns, 50 ns, 75 ns, and 100 ns) of the trajectory as references.

Molecular dynamics simulation was performed to explore the binding conformations and to analyze the stability of the inhibitors in binding with JAK1. The MD simulation of the most active compound 42 was carried out for 100 ns in explicit solvent. To analyze the binding interactions, an average protein–ligand complex structure was extracted from the last 2 ns of the simulation trajectory. The binding interactions between compound 42 and JAK1 are shown in Fig. 1a. Root mean square deviation (RMSD) analysis suggested that the system converged at around 25 ns into the simulation. The RMSD of the ligand with respect to the conformations at the initial 1st ns as well as the 25th ns, 50th ns, 75th ns, and 100th ns of the trajectory are shown in Fig. 1b. During the simulation, the pyrrolopyridine of compound 42 formed H-bond interactions with F958 and L959 at the hinge region of JAK1. The chlorobenzyl end of the compound was enclosed within the hydrophobic pocket under the P-loop and formed interactions with hydrophobic residues such as F886 and V889. This conformation of compound 42 was similar to the conformation of the selective JAK1 inhibitor (LKT) reported from the X-ray crystallography experiment by Su et al.^31^. The carboxamide moiety from pyrrolopyridine extended towards the bulk solved. Hydrophobic interactions were also observed between the inhibitor and the hydrophobic residues L881, F958, and L1010.

**Figure 2:**
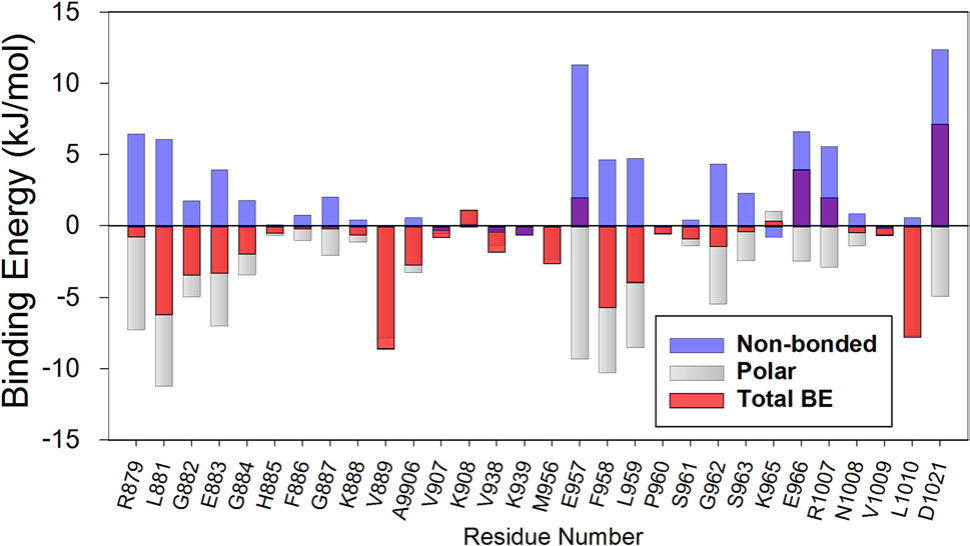
Residues that showed a high contribution to the total binding energy during the MD simulation of compound **42-JAK1** interaction. The energy values of non-bonded, polar, and total binding energy are shown in blue, grey, and red color respectively. Energy values are given in kJ/mol.

The BE of compound 42 was evaluated to estimate its binding affinity with JAK1 using the MM-PBSA method. Fifty snapshots of the protein–ligand complex were collected from the last 5 ns of the MD simulation trajectory and were used for the BE calculation. The most active compound was bound to JAK1 with a BE value of − 113.9 kJ/mol. The result of the BE calculation is shown in Table 4. The contribution of the Van der Waals, electrostatic, polar, and non-polar energy terms were  − 192.9 kJ/mol, − 42.7 kJ/mol, − 141 kJ/mol, and − 19.3 kJ/mol, respectively. Energy contribution analysis for individual residues showed that the residues V889, L1010, L881, F958, L959, G882, E883, A906, M956, K979 were among the highest contributors to the total BE with each residue contributing more than − 2 kJ/mol. The non-bonded energy term, polar energy term, and total binding energy of the binding site residues that made vital interactions with the most active compound 42 are shown in Fig. 2. The non-bonded energy term which includes both hydrophobic and electrostatic interactions was represented in blue color. Polar solvation energy was represented in grey color and the total binding energy was shown in red color. The Van der Waal energy term made the biggest contribution to the binding of the inhibitor. This observation from the BE calculation corroborates the interaction study which showed that compound 42 formed only two polar interactions while forming multiple hydrophobic interactions with JAK1. These results suggested that hydrophobic interaction could play an important role in the binding of the JAK1 inhibitor. For reference, we also evaluated the BE of Tofacitinib. Tofacitinib is an FDA-approved, pan JAK inhibitor that has been comprehensively studied and with structural data available in protein repository (PDB ID 3EYG). In the binding energy evaluation, Tofacitinib showed a high BE value of − 126 kJ/mol suggesting a tight binding with JAK1. To verify the simulation results of the compound 42-JAK1 interaction, the simulation of compound 42 was repeated and the results were compared with the results from the first simulation. In the second simulation of compound 42 (simulation 2), the total BE was − 110 kJ/mol. The analysis showed that the binding conformation of compound 42 was similar to that of the conformation observed in simulation 1. The comparison between the binding poses of compound 42 from the first and the second simulation is shown in Fig. S2a (Supplementary Material). The binding interaction of Tofacitinib with JAK1 from the MD simulation is shown in Fig. S2b (supplementary material).

### 3D-QSAR

**Figure 3:**
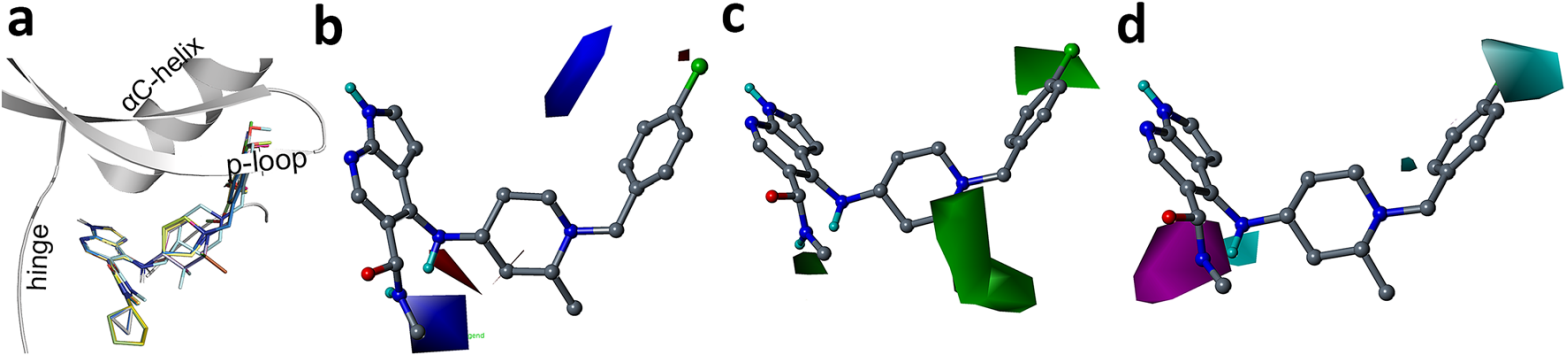
Alignment and contour maps from the CoMFA and CoMSIA models. (**a**) Alignment of the compounds inside the receptor. (**b**) Electrostatic contour map. Blue contour represents region favorable for electropositive substituents. (**c**) Steric contour map. Green contour represents a region favorable for bulky substituents. (**d**) Hydrophobic contour. Cyan contour represents regions favorable for hydrophobic substituents whereas, magenta color represent region favorable for non-hydrophobic substituents.

For the 3D-QSAR study, the dataset compounds were sketched and minimized in Sybyl X 2.1 (Tripos, St Louis, MO, USA). The alignment of the compounds was performed by superimposing the common substructure of the compounds with that of the structure of compound 42 from the MD study. The alignment of the compounds is shown in Fig. 3a. To develop a predictive model and validate its predictive ability, we used a stratified random sampling approach to split the dataset into a test set (15 compounds) and training set (35 compounds) following a 1:2 ratio. By repeating the random sampling, we generated five different sets of the training set and test set pairs (Sets 1–5). For each of the five sets, five CoMFA models were developed using the five charge schemes namely Gasteiger, Gasteiger-Hückel, Delre, Pullman, and MMFF94. The statistical results of the CoMFA models for the different charge schemes are shown in Table S1 (Supplementary material). Finally, a final model was selected based on high internal and external predictive ability and a low optimal number of components (ONC). The CoMFA model based on the Gasteiger charge scheme showed relatively better statistical values than the models based on other charge schemes. The selected CoMFA model showed internal predictive *q*^2^ values of 0.62 at an optimal number of component values of 3 and a non-validated *r*^2^ value of 0.82. When evaluated against the test set compounds, the model showed an $r_{pred}^{2}$ value of 0.86, suggesting that the model can predict the activity values reasonably. The statistical results of the CoMFA models are shown in Table 2.

**Table 2:** Statistical results of the CoMFA and CoMSIA models.

Parameters	CoMFA	CoMSIA
*q*^2^	0.62	0.52
ONC	3	3
SEP	0.45	0.52
*r*^2^	0.82	0.74
SEE	0.66	0.53
F value	48.39	30.08
BS *r*^2^	0.84	0.86
BS SD	0.22	0.30
Progressive scrambling (*Q*^2^)	0.54	0.52
$r_{pred}^{2}$	0.70	0.69
$Q_{F1}^{2}$	0.69	0.69
$Q_{F2}^{2}$	0.67	0.68
$Q_{F3}^{2}$	0.68	0.69
*CCC*	0.94	0.59
*MAE*	0.25	0.25
$r_{m}^{2}$	0.79	0.83
**Influence of different fields (%)**		
S	43	–
E	57	–
H	–	100

*q*^2^: cross-validated correlation coefficient; ONC: Optimal number of components; SEP: Standard Error of Prediction; *r*^2^: non-cross-validated correlation coefficient; SEE: Standard Error of Estimation; *F* value: F-test value; *r*^2^; BS-*r*^2^: Bootstrapping *r*^2^ mean; BS-SD: Bootstrapping Standard deviation; $r_{pred}^{2}$: predictive correlation coefficient; S: Steric; E: Electrostatic; *CCC*: Concordance correlation coefficient; More details on $Q_{F1}^{2}$*,* $Q_{F2}^{2}$,${ }Q_{F3}^{2}$, *MAE* and $r_{m}^{2}$ are given in Supplementary Material.

We also developed the CoMSIA models based on the training set and charge scheme used for the CoMFA model development. Several models were derived for each combination of the CoMSIA descriptors and the statistical results were evaluated (Table S2). The CoMSIA model based on hydrophobic descriptor with *q*^2^, *r*^2^, and *ONC* values of 0.52, 0.82, and 3 was selected since this model gave the highest external predictive value of 0.69. The statistical results of the CoMSIA models are shown in Table 2. The scatter plot between the actual and the predicted activity values from the CoMFA and CoMSIA models is shown in Fig. S3 (Supplementary Material). The analysis showed that the CoMFA and CoMSIA models were able to predict the activities of inhibitors with reasonable accuracy.

The CoMFA model showed a *Q*^2^ value of 0.54 while the CoMSIA model showed a slightly lower value of 0.52 in progressive scrambling. The predictive ability of the selected CoMFA and CoMSIA models were tested using various external validation techniques^1^ and the results are provided in Table 2. The CoMFA model showed ${ }Q_{F1}^{2}$, $Q_{F2}^{2}$, $Q_{F3}^{2}$, (*CCC*), *MAE*, and $r_{m}^{2}$ of 0.69, 0.67, 0.68, 0.94, 0.25 and 0.79 respectively. Similarly, The CoMSIA model showed $Q_{F1}^{2}$, $Q_{F2}^{2}$, $Q_{F3}^{2}$, (*CCC*), *MAE*, and $r_{m}^{2}$ of 0.69, 0.68, 0.69, 0.59, 0.25 and 0.83 respectively. These observations suggested that the CoMFA and CoMSIA models have reasonable predictive ability and reliability.

The williams plots for the CoMFA and CoMSIA models are provided in Fig. S6 (Supplementary materials). The predictions in both the models showed standard residual values between + 3 and − 3 and leverage values less than the threshold value of 0.25. These results indicated that the test set predictions are within the AD of the CoMFA and CoMSIA models. Hence, these predictions may be considered reliable.

### Contour map analysis

The contour map analysis of the CoMFA and CoMSIA maps was carried out to study the favorable and unfavorable sites for various chemical substitutions. The electrostatic and steric contour maps from the CoMFA model and the hydrophobic contour map from the CoMSIA model are shown in Fig. 3. Blue contours in the electrostatic map represent regions favorable to positive substitutions. The green contours in the steric contour map indicate regions favorable for bulky substitutions. Cyan and magenta color contours in the hydrophobic map represent hydrophobic favorable and hydrophilic favorable regions.

Blue contours near the carboxamide and also near the meta-position of the chlorobenzyl suggested that electropositive substituents in those positions could enhance the inhibitory activity against JAK1 (Fig. 3b). From the analysis, we observed that bulky substitutions at the chlorobenzyl moiety and near the methyl group of the methyl piperidine could improve the inhibitory activity against JAK1 (Fig. 3c). Hydrophobic substitutions near the chlorobenzyl could enhance the activity against JAK1 whereas non-hydrophobic substituents are favored near the carboxamide (Fig. 3d). Taken together, these contour maps provide an overview of the regions favorable for various chemical substitutions.

### Designing new compounds

Following the contour map analysis, an inhibitor design scheme was developed as shown in Table 3. Up to 150 compounds were designed by adding substituents based on the design scheme. The inhibitory activities of the designed compounds were predicted using the selected CoMFA and CoMSIA models and seventeen compounds that showed a higher predicted activity value than the most active compound (42) in the dataset were further analyzed. The structure and the predicted activity values of the seventeen designed compounds are given in Table 3.

**Table 3:**
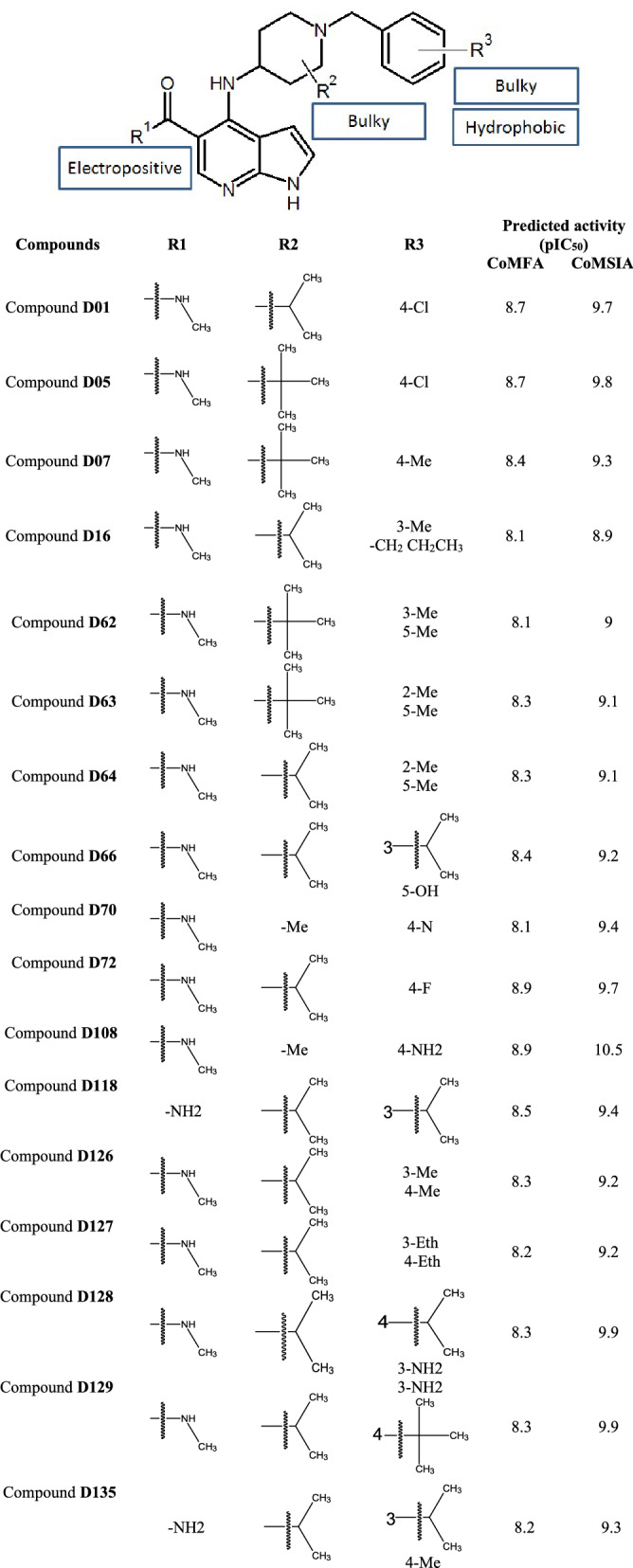
Structure of the designed compounds along with the predicted pIC_50_ values.

The interaction study from MD simulation and the contour maps were co-analyzed to explore the structural features necessary to improve the activity values against JAK1. Some of the compound fragments which were used as substituents were collected from published literature while some were taken from the in-house compound library. We performed the designing of new compounds by building upon the most active compound 42 as shown in Table 3. As a first step, we substituted the R^1^ and R^2^ with electropositive and bulky substituents while keeping the Cl substituent at the R^3^ position unchanged. The 3D-QSAR prediction showed that the electropositive and bulky substituents at R^1^ and R^2^ improved the activity values as observed in compounds D01 and D05. Next, we also examined the R^3^ position by adding bulky and hydrophobic substituents with three or four carbon atoms. The prediction showed that the substitution of bulky and hydrophobic substituents increased the activity values as seen in D129, D135, D62, etc. When we substituted the R^3^ position with 3-NH2 or 4-NH2, we found the activity values of the compounds increased considerably higher than the compounds with methyl or ethyl groups as seen in compounds D108, D128, and D129. In D70 and D72, we have also substituted the Cl atom of the compound D01 and D05 with other heavy atoms such as N, and F atoms which increased the activity values. We also substituted the R^3^ position with aromatic rings such as five and six-membered rings, which showed a decrease in the activity value. Similarly, aromatic substituents in R^1^ and R^2^ positions also lead to a decrease in activity values. Hence we deduce that, though bulky substituents are favorable at R^1^, R^2^, and R^3^, very large substituents are not favorable. The SMILES structures of the 150 compounds were provided in Table S5 (supplementary material).

**Figure 4:**
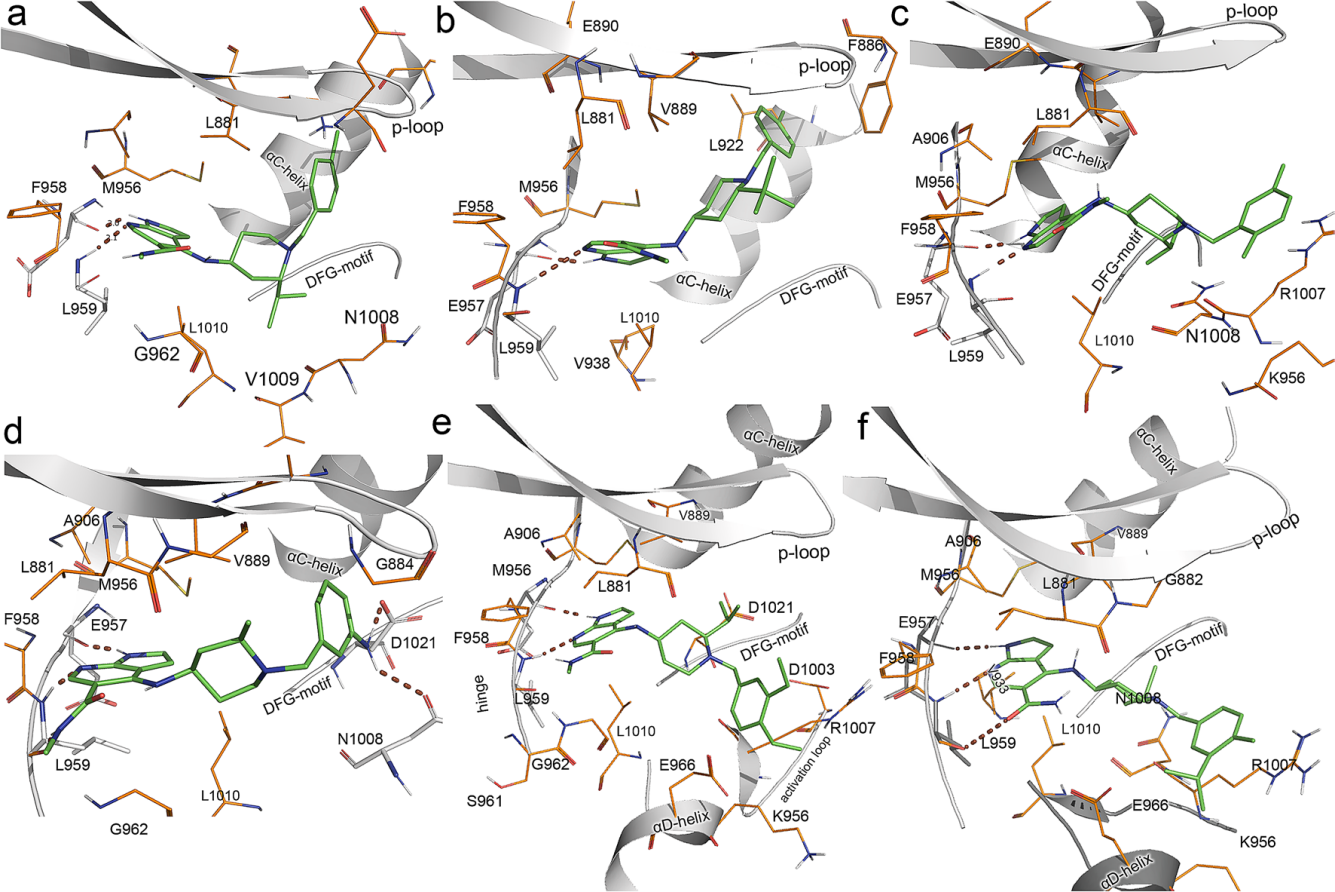
H-bond interactions of the designed compounds with JAK1 from the MD simulation. (**a**) D01-JAK1 interactions, (**b**) D07-JAK1 interactions, (**c**) D64-JAK1 interactions, (**d**) D108-JAK1 interactions, (**e**) D127-JAK1 interactions, (**f**) D135-JAK1 interactions. The inhibitors are shown in green color stick representations. H-bond interactions are represented by brown dotted lines and residues forming H-bonds are shown in grey stick representations. Residues that formed hydrophobic interactions are shown in orange color lines.

Molecular dynamics simulation and BE calculation of the seventeen designed compounds were carried out to study the binding interactions and the binding affinity with JAK1. The binding energy values are shown in Table 4. The binding interactions of compounds D01, D07, D64, D108, D127, and D135 showed higher binding affinity than the reference compound Tofacitinib (− 126 kJ/mol) and are further analyzed and shown in Fig. 4. Of the selected design compounds, compound D127 showed the highest binding affinity with a binding energy value of − 137 kJ/mol (Fig. 4e).

**Table 4:** The energy contribution of the various energetic terms (van der Waals energy, electrostatic energy, polar solvation energy, and non-polar solvation energy/SASA) to the total binding energy.

Compounds	Van der Waals (kJ/mol)	Electrostatics (kJ/mol)	Polar solvation (kJ/mol)	SASA (kJ/mol)	Total binding energy (kJ/mol)
Compound 42 (most active compound)	− 192.9	− 42.7	141.0	− 19.3	− 113.9
Tofacitinib	− 205	− 74.2	170.5	− 17.3	− 126
**Designed compounds**					
Compound **D01**	− 230.4	− 51.1	177.4	− 23.6	− **127.2**
Compound **D05**	− 210.8	− 40.9	150.8	− 21.3	− 122.2
Compound **D07**	− 225.2	− 40.9	162.2	− 22.0	− **125.9**
Compound **D16**	− 217.3	− 47.8	162.5	− 21.0	− 124.2
Compound **D62**	− 184.5	− 46.3	135.1	− 20.0	− 115.7
Compound **D63**	− 203.5	− 45.7	151.3	− 20.4	− 118.2
Compound **D64**	− 202.2	− 50.3	145.4	− 19.5	− **126.6**
Compound **D66**	− 209.2	− 78.0	193.9	− 22.3	− 115.6
Compound **D70**	− 232.7	− 55.9	188.0	− 23.3	− 123.8
Compound **D72**	− 198.7	− 53.5	148.9	− 19.2	− 122.5
Compound **D108**	− 214.0	− 69.8	175.7	− 21.3	− **129.4**
Compound **D118**	− 201.6	− 38.5	147.2	− 22.1	− 115.0
Compound **D126**	− 205.8	− 54.8	160.4	− 20.8	− 120.9
Compound **D127**	− 200.2	− 43.1	127.0	− 20.9	− **137.2**
Compound **D128**	− 224.0	− 55.4	185.7	− 23.8	− 117.5
Compound **D129**	− 206.1	− 51.3	166.3	− 21.9	− 113.1
Compound **D135**	− 218.1	− 50.9	164.2	− 21.5	− **126.3**

The energy values are given in kJ/mol.

The compound D01 (Fig. 4a), like compound 42, has a chlorine atom at the benzyl moiety and formed a conformation similar to that of compound 42. The pyrrolopyridine moiety formed H-bond interactions with E957 and L959. The tert-butyl moiety from the piperidine extended out into the bulk solvent while the chlorobenzyl moiety folded into the hydrophobic pocket. The compound D07 (Fig. 4b), formed H-bond with the E957 and L959 at the hinge region while, the benzyl moiety extended into the hydrophobic pocket, similar to compound 42. Compound D07 which has a methyl group at the benzyl moiety also exhibited a similar binding pattern with 42 and folded in-ward into the hydrophobic pocket. Compound D64 also formed H-bond interactions with the hinge at E957 and L959. However, the dimethyl benzyl moiety extended out of the hydrophobic pocket and formed non-bonded interactions with K956, R1007, and N1008 (Fig. 4c). The compound D108, which possessed an amine group at the benzyl moiety, also folded toward the hydrophobic pocket. D108 formed three H-bond interactions at the hinge with E957 and L959. The amine group at the benzyl formed H-bond interaction with N1008 at the activation loop and D1021 at the DFG motif. The methyl group at the piperidine extended up and out towards the glycine-rich loop (Fig. 4d). Interaction studies of the D127-JAK1 interaction in Fig. 4e showed that the pyrrolopyridine of D127, like compound 42, was able to form H-bond interaction at the hinge with E957 and L959. However, the diethyl benzyl moiety of compound D127 extended outward of the hydrophobic pocket and formed non-bonded interactions with E966 and K965 of the αD-helix and R007 of the activation loop. The H-bond interactions with the hinge residues were also observed in all the designed compounds. The compound D135 which showed the third-highest binding affinity with a BE value of − 126 kJ/mol showed an additional H-bond interaction with Leu959 at the hinge (Fig. 4f). In addition to the non-bonded interactions at the αD-helix, the compound D135 also interacted with the R1007 via a cation-pi interaction. The energy values of the individual residues that make significant contributions are given in Table S4 (Supplementary Materials). The interactions of the compounds D05, D16, D62, D63, D66, D70, D72, D118, D126, D128, and D129 showed binding energy values lower than − 113 kJ/mol are given in Fig. S4 (Supplementary Materials). The ligand and protein RMSD for all the compounds are given in Fig. S5 (Supplementary Materials).

## Discussion

The dataset compounds shared a common substructure consisting of a fused pyrrolopyridine and a benzyl moiety which are linked together by either piperidine, a pyrrolidine, an azabicyclo[3.2.1]octane, or an azepane ring. The pyrrolopyridine moiety, as seen in the most active compound 42, was anchored at the hinge while the piperidine was bound between the activation loop and glycine-rich loop through hydrophobic interactions. The benzyl end (chlorobenzyl in compound 42) of the compound took an inward turn and occupied the hydrophobic pocket formed by residues from the P-loop, the αC-helix, and the activation loop.

The conformational analysis of compounds D01, D07, D64, D108, D127, and D135 showed that these designed compounds were able to bind to the hinge via H-bond interactions with the E957 and L959. This interaction with the hinge is considered to be important for anchoring the inhibitors inside the kinase^32,33^. However, the benzyl end of the designed compounds adopted either a fold-in conformation, or a fold-out conformation. The designed compounds D01 and D05 adopted the inward fold-in conformation in which the benzyl moiety occupied the hydrophobic pocket, similar to the observation in compound 42 (most active compound). On the other hand, the compounds D64, D127, and D135 adopted the fold-out conformation and extended the substituted benzyl end toward the bulk solvent. This fold-out conformation is reminiscent of the conformation of the selective inhibitor EYQ –JAK1 interaction (PDB ID 6GGH). This conformation contradicts the observation made in compound 42-JAK1 interaction (Fig. 1a) in which the chlorobenzyl folded into the hydrophobic pocket. The reason behind the difference in the conformation of the designed compounds could not be verified. However, further analysis of compounds D64, D108, and D135 showed that all these compounds possessed bulky substituents at the benzyl ring and adopted the fold-out conformation. The bulky substituents at the benzyl may cause steric clash at the hydrophobic pocket in a fold-in conformation, while the fold-out conformation may allow non-bonded interactions with residues from the αD-helix and the activation loop. These observations suggested that compounds with small substituents tend to occupy the hydrophobic pocket whereas the compounds with bulky substituents tend to extend outward. Earlier computational studies by our group and also by other experimental researchers have shown that, while the interactions with the hinge of kinases are stable, the interactions at the hydrophobic pocket are more flexible^21,34–36^. This flexibility allows for variation in inhibitor binding and is considered to be a mechanism for selective inhibition. The high binding affinity with JAK1 and their unique binding conformation make these compounds interesting potential candidates for JAK1 inhibition. Further assessment through experimental studies is essential to verify the potential of the designed compounds.

Taken together, the protein–ligand interaction studies suggested that these pyrrolopyridine based JAK1 inhibitors can adopt two distinct conformations. The structure–activity analysis revealed vital structural properties to improve inhibitory activity against JAK1. At least six of the compounds showed a higher binding affinity against JAK1 as compared to the approved JAK1 inhibitor Tofacitinib. The outcome of this study could provide vital information for the development of more potent JAK1 inhibitors.

## Supplementary Information

Supplementary Information.

## Data Availability

All relevant data are contained within the manuscript and the supplementary material. Additional raw data will be available upon request.
