# Supplementary material for: Computer aided designing of novel pyrrolopyridine derivatives as JAK1 inhibitors
Source: Sci Rep. 2021 Nov 29;11:23051. doi: 10.1038/s41598-021-02364-2 (PMC8630053; doi:10.1038/s41598-021-02364-2)
Supplement: Supplementary file 1 — Supplementary Information. [file 41598_2021_2364_MOESM1_ESM.pdf]

Computer Aided Designing of Novel Pyrrolopyridine Derivatives as JAK1 Inhibitors

Seketoulie Keretsu[1], Suparna Ghosh[1], Seung Joo Cho[1,2,*]

[1]*Department of Biomedical Sciences, College of Medicine, Chosun University, Gwangju 501-759, Republic of Korea*

[2]*Department of Cellular·Molecular Medicine, College of Medicine, Chosun University, Gwangju 501-759, Republic of Korea*

Supplementary Materials

Figure S1. Binding pose of inhibitors inside JAK1 from MD study **(a).** The overlap between the docked pose and the crystal pose of Tofacitinib. **(b)** The binding of Compound 42 with JAK1 as observed from the molecular docking study. The H-bond interactions are shown in brown dotted lines. Residues that formed hydrophobic interactions are shown in orange color stick representation

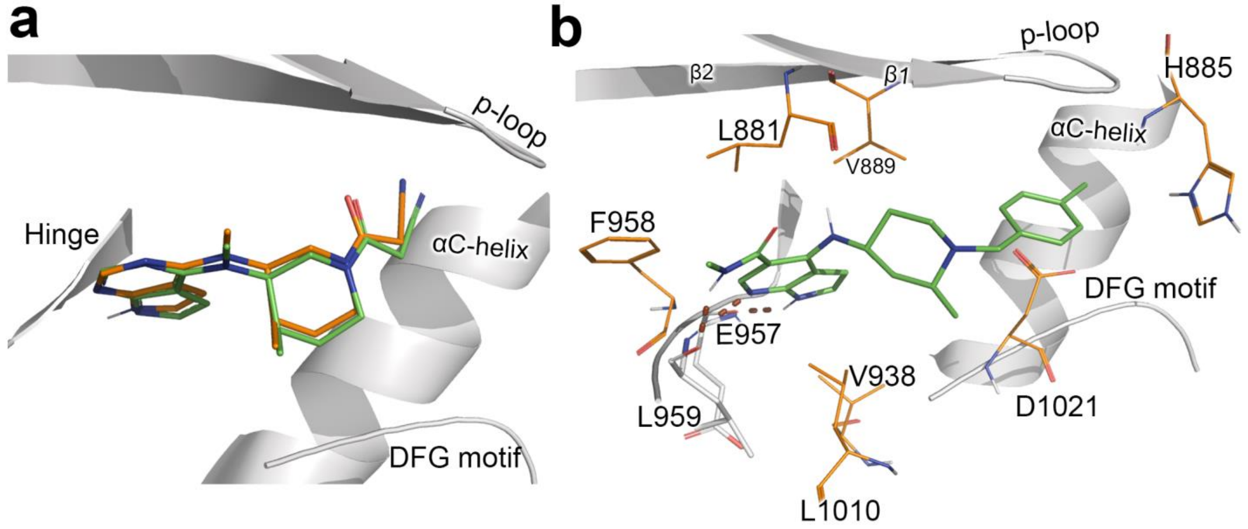

Figure S2. Binding pose of inhibitors inside JAK1 from MD study **(a)** The overlap between the compound 42 from the two molecular dynamics simulations. The structure of compound 42 from the first simulation is given in green color while the pose from the second simulation is given in orange color. **(b)** Binding interactions between the reference compound Tofacitinib and JAK1. The H-bond interactions are shown in brown dotted lines. Residues that formed hydrophobic interactions are shown in orange color stick representation.

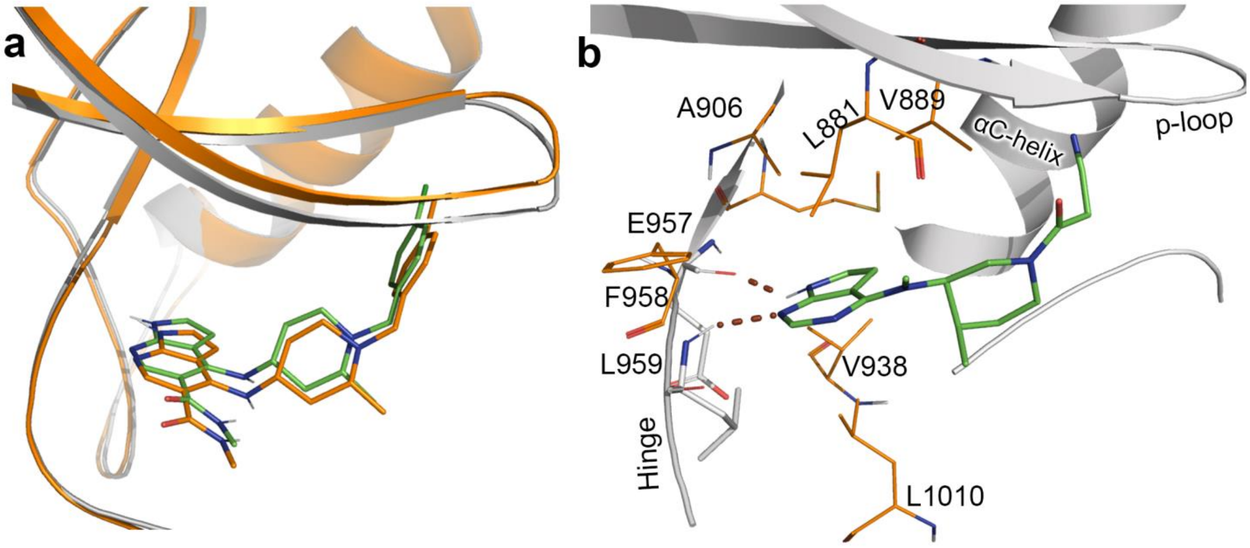

Figure S3. Scatter plot of the actual pIC50 and the predicted pIC50 values of the compounds from the CoMFA and CoMSIA models.

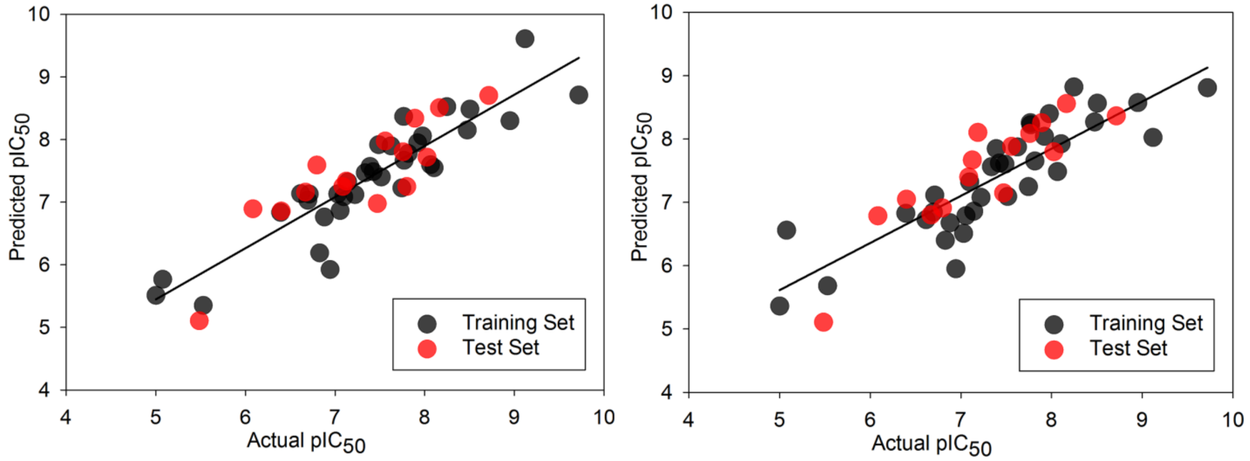

Figure S4. Binding interactions of the designed compounds which showed higher binding energy value than compound 42 with JAK1. **(a)** D05-JAK1 **(b)** D16-JAK1 **(c)** D62-JAK1 **(d)** D63-JAK1 **(e)** D66-JAK1 **(f)** D70-JAK1 **(g)** D72-JAK1 **(h)** D118-JAK1 **(i)** D126-JAK1 **(j)** D128-JAK1 **(k)** D129-JAK1

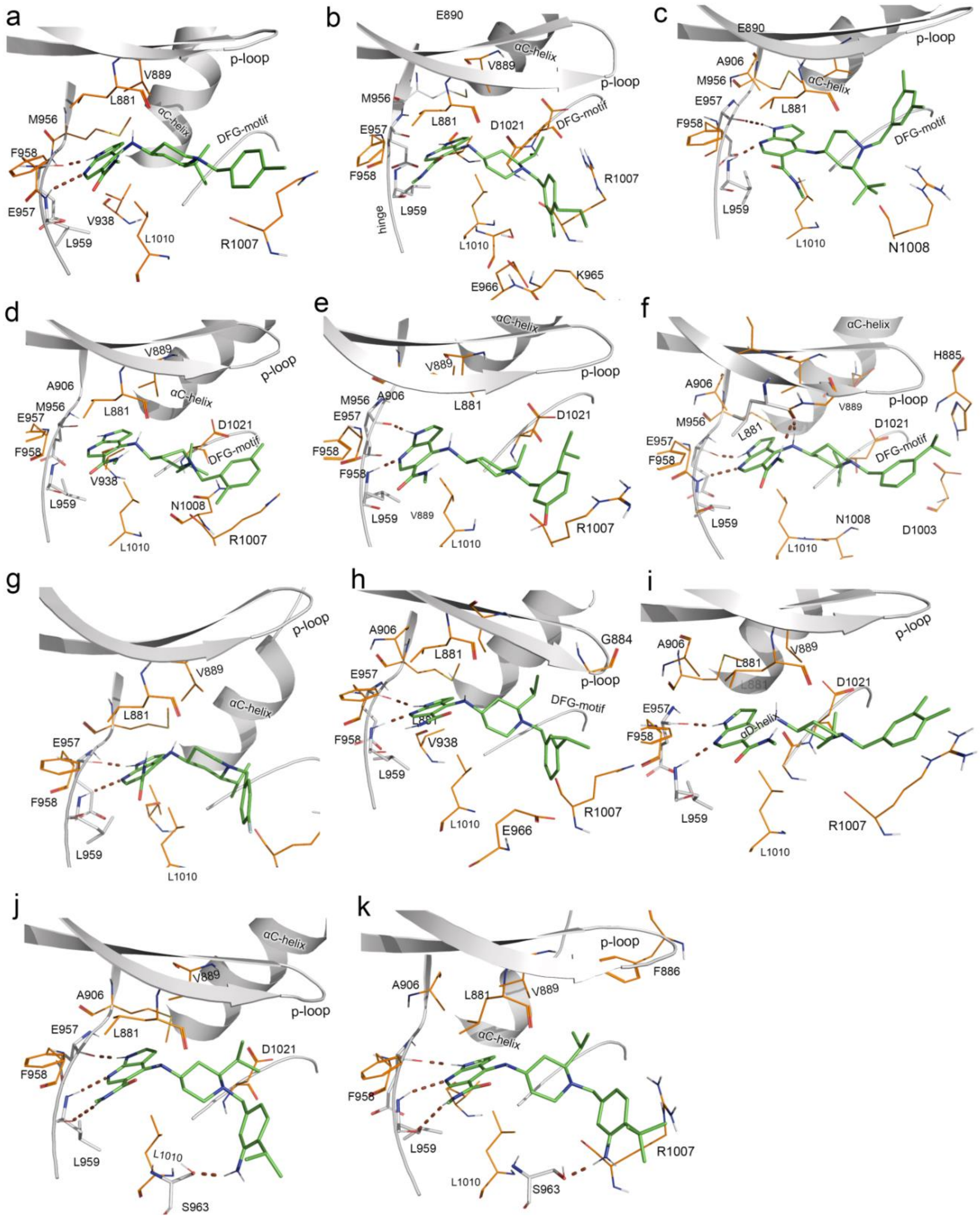

Figure S5. RMSD of the designed compounds and JAK1 during molecular dynamics simulations. The RMSD of the inhibitors are shown in black color while the RMSD of JAK1 during the simulation is shown in red color.

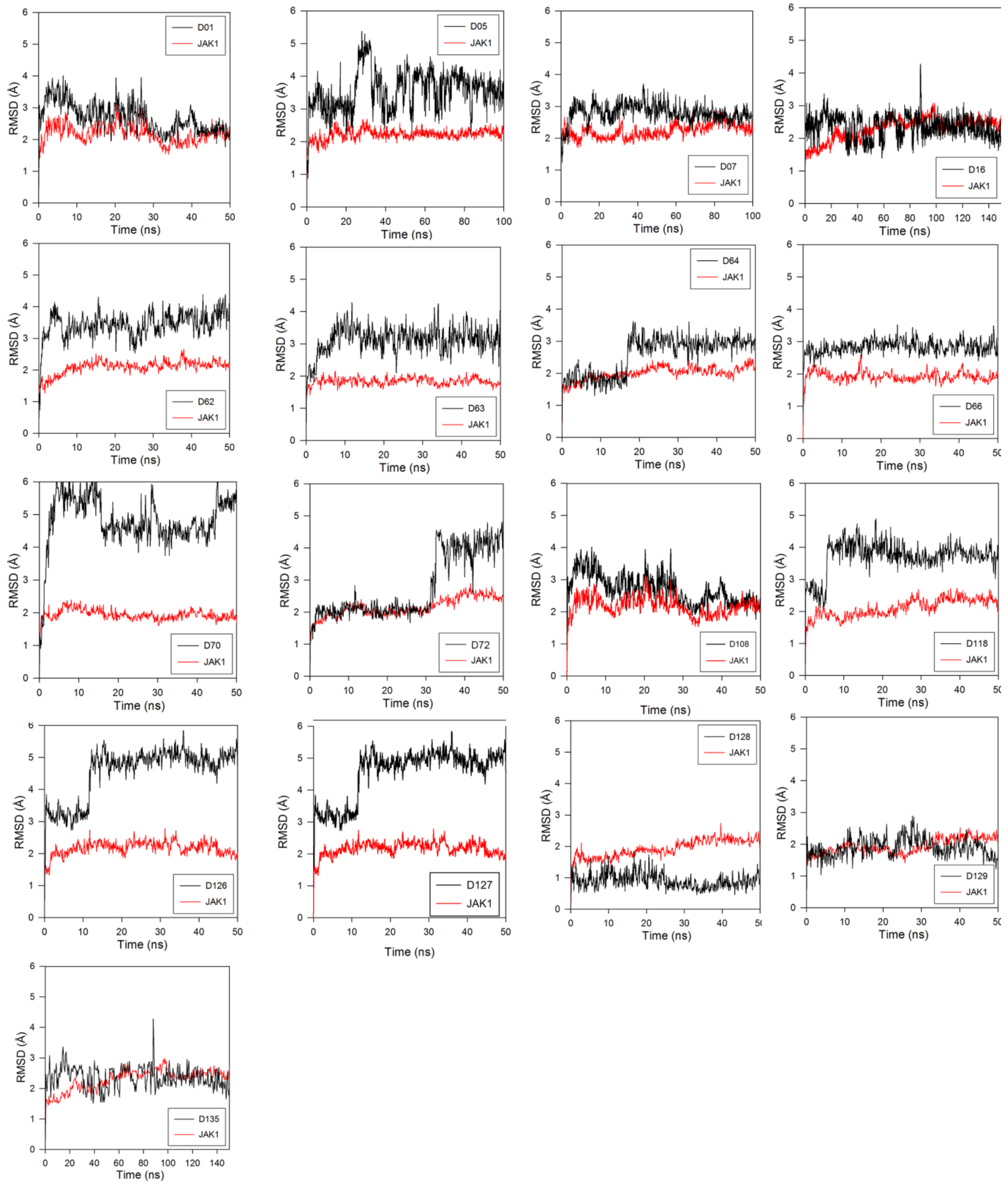

Figure S6. Applicability Domain analysis. (a) CoMFA and (b) CoMSIA. The threshold limits for the standard residuals and leverage are shown in blue and red colors lines respectively. The leverage threshold value for both models is 0.25. Test set and training set compounds are shown in red and black color respectively.

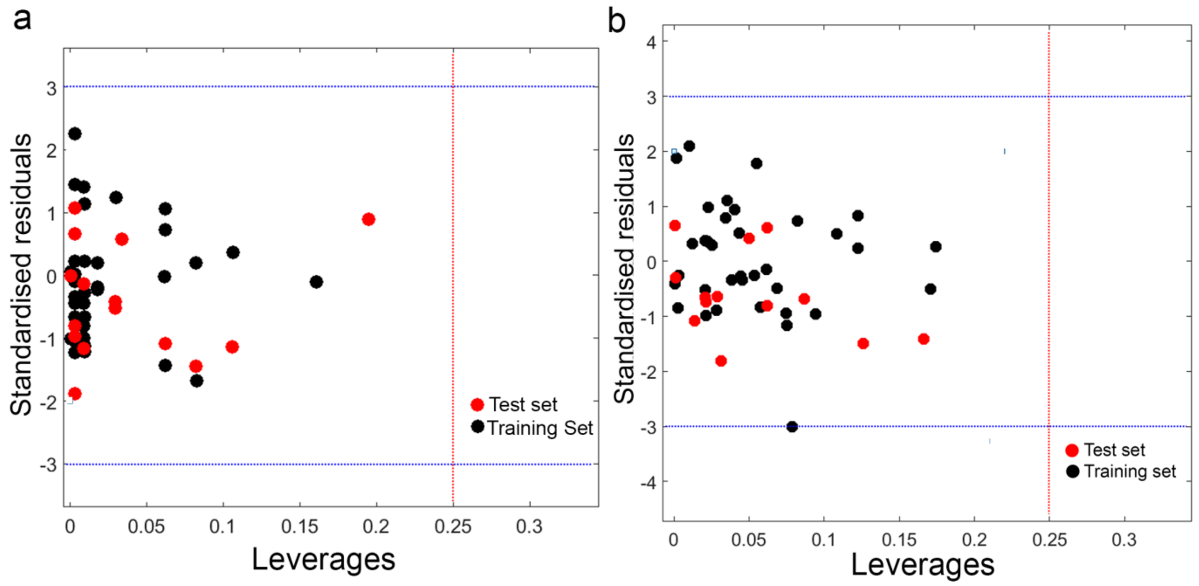

Tables

Table S1. Statistical results of the various CoMFA models**.** The Set 1-5 represents the training set and test set pairs that resulted from the stratified random sampling.

Set 1	Gasteiger	Gasteiger-Hückel	Delre	Pullman	MMFF94
q^2	**0.62**	0.6	0.59	0.59	0.59
SEE	**0.66**	0.68	0.68	0.68	0.68
NOC	**3**	3	3	3	2
r^2	**0.82**	0.84	0.85	0.84	0.79
SEP	**0.45**	0.43	0.41	0.42	0.47
F-value	**48.39**	54.58	58.35	55.31	63.2
External r^2_{pred}	**0.86**	0.79	0.8	0.76	0.81
		**Percentage Contribution**			
Steric	**43**	52	45	57	45
Electrostatic	**57**	48	55	43	55

Set 2	Gasteiger	Gasteiger-Hückel	Delre	Pullman	MMFF94
q^2	0.67	0.66	0.67	0.68	0.64
SEE	0.53	0.53	0.52	0.51	0.53
NOC	4	4	3	3	2
r^2	0.89	0.91	0.88	0.87	0.82
SEP	0.3	0.285	0.31	0.32	0.38
F-value	62.14	72.38	79.21	71.3	74.68
External r^2_{pred}	0.59	0.64	0.65	0.67	0.68
		**Percentage Contribution**			
Steric	50	53	45	62	52
Electrostatic	50	47	55	38	48

Set 3	Gasteiger	Gasteiger-Hückel	Delre	Pullman	MMFF94
q^2	0.56	0.56	0.56	0.55	0.55
SEE	0.7	0.7	0.72	0.7	0.72
NOC	5	4	6	4	5
r^2	0.89	0.86	0.9	0.88	0.9
SEP	0.35	0.36	0.33	0.36	0.33
F-value	46.23	48.11	44.74	56.47	52.64
External r^2_{pred}	0.66	0.67	0.55	0.68	0.52
		**Percentage Contribution**			
Steric	47	54	46	60	52
Electrostatic	53	46	54	40	48

Set 4	Gasteiger	Gasteiger-Hückel	Delre	Pullman	MMFF94
**q^2**	0.61	0.58	0.6	0.6	0.57
**SEE**	0.66	0.68	0.68	0.67	0.68
**NOC**	3	3	4	3	2
**r^2**	0.86	0.85	0.89	0.86	0.82
**SEP**	0.39	0.41	0.36	0.39	0.44
**F-value**	65.42	57.6	58.82	64.52	74.56
**External r^2_{pred}**	0.5	0.45	0.5	0.53	0.55
		**Percentage Contribution**			
**Steric**	43	54	42	55	50
**Electrostatic**	57	46	58	45	50

Set 5	Gasteiger	Gasteiger-Hückel	Delre	Pullman	MMFF94
**q^2**	0.66	0.71	0.688	0.68	0.69
**SEE**	0.6	0.53	0.57	0.58	0.56
**NOC**	6	4	6	6	6
**r^2**	0.91	0.88	0.92	0.92	0.91
**SEP**	0.3	0.33	0.29	0.29	0.29
**F-value**	48.29	59.68	5094	52.45	5088
**External r^2_{pred}**	0.43	0.5	0.54	0.56	0.57
		**Percentage Contribution**			
**Steric**	46	50	43	59	51
**Electrostatic**	54	49	57	41	49

q^2: cross-validated correlation coefficient; ONC: Optimal number of components; SEP: Standard Error of Prediction; r^2: non-cross-validated correlation coefficient; SEE: Standard Error of Estimation; F value: F-test value; r^2_{pred}: predictive correlation coefficient; S: Steric; E: Electrostatic;

Table S2. The statistical results of the selected CoMSIA model.

CoMSIA	q^2	ONC	SEP	r^2	SEE	F value	Percentage contribution				
							**S**	**E**	**H**	**A**	**D**
S	0.49	3	0.75	0.76	0.52	32	100	-	-	-	-
E	0.47	6	0.8	0.82	0.48	20.17	-	100	-	-	-
**H**	**0.52**	**3**	**0.54**	**0.82**	**0.53**	**30.08**	-	-	**100**	-	-

A	0.01	2	1.04	0.1	0.97	2.3	-	-	-	100	-
D	0.46	4	0.78	0.63	0.65	12.82	-	-	-	-	100
SE	0.52	4	0.74	0.81	0.46	32	26	74	-	-	-
EH	0.49	3	0.75	-	-	-	-	-	-	-	-
EA	0.46	4	0.78	-	-	-	-	-	-	-	-
ED	0.48	5	0.78	-	-	-	-	-	-	-	-
SH	0.45	3	0.78	-	-	-	-	-	-	-	-
SA	0.42	4	0.81	-	-	-	-	-	-	-	-
SD	0.54	5	0.73	0.81	0.47	25.1	58	-	-	-	42
HA	0.48	3	0.75	-	-	-	-	-	-	-	-
HD	0.53	4	0.73	0.75	0.52	23.59	-	-	67		33
SEH	0.51	3	0.73	0.8	0.46	42.73	16	56	28	-	-
SEA	0.50	4	0.75	0.82	0.45	34.62	24	70	-	5	-
SED	0.53	5	0.74	0.83	0.44	28.87	18	59	-	-	23
EHA	0.48	3	0.75	-	-	-	-	-	-	-	-
EHD	0.51	4	0.74	-	-	-	-	-	-	-	-
SHA	0.43	2	0.79	-	-	-	-	-	-	-	-
SHD	0.53	4	0.73	0.78	0.5	26	29	-	43	-	28
EAD	0.48	5	0.78	-	-	-	-	-	-	-	-
HAD	0.5	4	0.77	0.75	0.53	23.16	-	-	57	10	33
SEHD	0.52	4	0.73	0.84	0.42	41.5	8	47	28	-	17

SEHA	0.51	3	0.74	0.80	0.46	43.24	16	54	27	3	-
SEAD	0.51	4	0.74	0.8	8	30.00	15	53	-	4	25
EHAD	0.5	4	0.75	0.8	0.47	30.26	-	50	22	4	23
SHAD	0.51	4	0.74	0.78	0.5	26.56	27	-	39	8	26
SEHAD	0.51	4	0.74	0.81	0.45	33.41	44	13	18	3	20

q^2: cross-validated correlation coefficient; ONC: Optimal number of components; SEP: Standard Error of Prediction; r^2: non-cross-validated correlation coefficient; SEE: Standard Error of Estimation; F value: F-test value; r^2_{pred}: predictive correlation coefficient; S: Steric; E: Electrostatic; H: Hydrophobic; A: Acceptor; D: Donor.

Table S3. The predicted activity values (pIC50) and the residual values of the compounds from the selected CoMFA and CoMSIA models.

**Compound**	**Actual pIC_{50}**	**CoMFA Predicted pIC_{50}**	**Residual**	**Actual pIC_{50}**	**CoMSIA (H) Predicted pIC_{50}**	**Residual**
1	7.06	6.89	0.17	7.06	6.81	0.25
2	6.71	7.15	-0.44	6.71	7.13	-0.42
3	7.19	7.80	-0.61	7.19	8.12	-0.93
4	6.39	6.86	-0.46	6.39	6.85	-0.45
5*	7.74	7.25	0.50	7.74	7.26	0.48
6*	6.69	7.05	-0.35	6.69	6.86	-0.17
7	5.08	5.79	-0.71	5.08	6.00	-0.92
8*	7.09	7.27	-0.18	7.09	7.42	-0.33
9	6.94	5.95	1.00	6.94	5.97	0.97
10	5.53	5.37	0.15	5.53	5.70	-0.17
11	5.00	5.53	-0.53	5.00	5.39	-0.39

12	5.48	5.13	0.36	5.48	5.13	0.35
13	6.80	7.61	-0.81	6.80	6.93	-0.13
14	6.67	7.18	-0.51	6.67	6.82	-0.15
15*	6.61	7.15	-0.54	6.61	6.75	-0.13
16*	7.14	7.34	-0.20	7.14	6.88	0.26
17	6.88	6.78	0.10	6.88	6.70	0.18
18	7.03	7.15	-0.12	7.03	6.53	0.50
19	6.40	6.88	-0.48	6.40	7.07	-0.67
20*	7.22	7.14	0.08	7.22	7.10	0.13
21	7.10	7.10	-0.01	7.10	7.34	-0.24
22	6.08	6.91	-0.83	6.08	6.81	-0.72
23*	6.83	6.21	0.62	6.83	6.42	0.41
24	7.13	7.35	-0.23	7.13	7.68	-0.56
25	7.39	7.59	-0.20	7.39	7.86	-0.47
26	7.76	7.82	-0.06	7.76	8.10	-0.34
27*	7.34	7.49	-0.15	7.34	7.58	-0.24
28	7.47	7.00	0.48	7.47	7.17	0.31
29	7.82	7.79	0.02	7.82	7.67	0.15
30	7.42	7.50	-0.08	7.42	7.64	-0.21
31*	8.03	7.73	0.29	8.03	7.81	0.21
32	7.92	7.96	-0.04	7.92	8.06	-0.13
33*	8.07	7.61	0.45	8.07	7.50	0.56
34	7.52	7.42	0.10	7.52	7.11	0.41
35*	7.49	7.93	-0.45	7.49	7.62	-0.13
36	7.97	8.07	-0.10	7.97	8.41	-0.44

37*	8.95	8.31	0.64	8.95	8.59	0.36
38	8.25	8.54	-0.29	8.25	8.83	-0.58
39	8.47	8.16	0.31	8.47	8.28	0.19
40*	7.56	7.99	-0.43	7.56	7.90	-0.34
41	8.50	8.50	0.01	8.50	8.58	-0.07
42	9.72	8.72	1.00	9.72	8.82	0.90
43	7.89	8.35	-0.46	7.89	8.27	-0.38
44*	7.62	7.91	-0.29	7.62	7.89	-0.27
45	8.71	8.72	0.00	8.71	8.38	0.34
46	8.16	8.52	-0.36	8.16	8.57	-0.41
47	7.76	8.38	-0.62	7.76	8.27	-0.51
48*	7.77	7.68	0.09	7.77	8.24	-0.47
49	8.10	7.56	0.54	8.10	7.94	0.17
50	9.12	9.62	-0.50	9.12	8.40	0.72

Test set compounds were marked with *.

Table S4. Energy contribution of the 30 residues with high contribution to the total Binding Energy are shown for interaction of JAK1 with selected compounds with higher binding affinity than Tofacitinib namely, compound 42 (most active compound), D01, D07, D64, D108, D127,and D135. Energy values are in kJ/mol.

	**Compound 42**	**D01**	**D07**	**D64**	**D108**	**D127**	**D135**
**V889**	-8.47	-7.65	-9.49	-4.85	-11.30	-6.13	-6.31
**L1010**	-7.66	-8.47	-8.50	-11.73	-5.76	-8.78	-10.70
**L881**	-6.08	-3.84	-1.64	-5.01	-6.17	-6.20	-5.29
**F958**	-5.58	-6.48	-4.13	-6.53	-5.57	-8.09	-6.06
**L959**	-3.85	-5.50	-3.52	-1.61	-6.45	-5.13	-3.39
**G882**	-3.36	-2.18	-2.29	-1.20	-1.38	-1.06	-2.19

**E883**	-3.23	-2.43	0.24	-0.34	-0.80	-1.50	1.88
**A906**	-2.64	-2.97	-1.45	-2.77	-3.34	-3.39	-3.26
**M956**	-2.55	-3.87	-4.20	-2.72	-2.39	-2.28	-2.41
**K970**	-2.18	0.61	-1.17	-1.28	-1.71	-0.26	-1.08
**G884**	-1.88	-1.38	-1.93	-0.17	-1.74	-1.19	-0.22
**V938**	-1.74	-0.97	-3.00	-1.64	-1.05	-1.54	-1.67
**E890**	-1.69	-2.55	-2.66	-1.85	-1.65	-2.34	-1.46
**G962**	-1.37	-3.25	-0.56	-1.61	-4.55	-1.36	-0.24
**E925**	-1.18	-0.62	-0.59	0.44	-1.80	-2.04	0.47
**K1018**	-1.17	0.65	-0.17	-1.00	-0.83	0.55	-0.43
**K974**	-0.95	0.49	-0.58	-0.90	-0.19	0.31	-0.40
**R893**	-0.83	0.64	-0.46	-0.58	-0.09	0.31	-0.16
**S961**	-0.80	-1.96	-0.38	-1.08	-1.81	-1.21	-0.65
**V907**	-0.70	-0.77	-0.81	-0.67	-0.62	-0.65	-0.55
**R879**	-0.68	5.03	-1.51	2.30	-0.07	1.12	1.14
**S909**	-0.64	-0.69	-2.73	-0.07	-1.62	-0.22	-0.05
**L891**	-0.64	-0.60	-0.32	-0.65	-0.91	-0.73	-0.58
**K941**	-0.60	0.65	0.51	-0.37	-0.19	0.78	0.02
**V1009**	-0.56	-2.41	-0.54	-1.13	-0.49	-1.07	-1.07
**K888**	-0.55	0.02	-1.85	0.39	-1.51	1.01	0.12
**K939**	-0.54	-0.30	-0.75	-0.42	-0.16	0.60	0.03
**P960**	-0.43	-0.50	-0.51	-0.69	-0.09	-0.66	-0.56
**H885**	-0.41	-0.75	-0.44	-0.08	-1.95	-0.11	-0.12
**V905**	-0.39	-0.37	0.09	-0.32	-0.50	-0.52	-0.39

Methodology:

Binding Energy Calculation:

The free energy of binding of the ligand to the receptor is given by the equation given below.

$$\Delta G_{binding} = \Delta G_{complex} - \left[\Delta G_{receptor} + \Delta G_{ligand}\right]$$

Where, $\Delta G_{complex}$, $\Delta G_{receptor}$ and ΔG_{ligand} are the free energies of the ligand-receptor complex, unbound receptor, and ligand respectively. The values of $\Delta G_{complex}$, $\Delta G_{receptor}$ and ΔG_{ligand} given in equation 1 are calculated based on equation 2 below.

$$\Delta G_{solvated} = E_{gas} + \Delta G_{solvation} - TS_{solute}$$

Where, $\Delta G_{solvated}$ is the free energy of the molecule (complex or receptor or ligand). E_{gas} represents the molecular mechanical energies and $\Delta G_{solvation}$ represents the solvation free

energies. *TS* represents the entropic contribution. The solvation free energies ($\Delta G_{solvation}$) can be further decomposed into electrostatics and nonpolar contributions. The solvation free energy was calculated using the Generalized Born (GB) method with non-polar solvation terms calculated based on the solvent-accessible surface area (SASA).

Validation techniques:

The bootstrapping method was used to evaluate the robustness of the model. The sensitivity of the model to chance correlation was tested using the predictive scrambling method (predictive scrambling *Q2*). To evaluate the predictive ability of the models against the external dataset, the r^2_{pred}, Q^2_{F1}, Q^2_{F2}, Q^2_{F3}, Concordance Correlation Coefficient (*CCC*), mean absolute error (*MAE*), and r^2_m validations were performed [1].

To test the predictive ability of the model against an external dataset, we calculated r^2_{pred} as:

$$\mathrm{r^2_{pred}} = {(\mathrm{SD} - \mathrm{PRESS})}/{\mathrm{SD}}$$

Where *SD* is the standard deviation between the activity value of the test set compounds and the mean activity value of the training set compounds. PRESS represents the sum of the square deviation between the predicted and the actual activity value of each compound in the test set.

The external Q2 functions to assess the external predictive performance of the 3D-QSAR models were calculated as shown below [2]:

$$\mathrm{Q^2_{F1}} = 1 - \frac{\sum_{i=1}^{n_{EXT}}(y_i - \hat{y}_i)^2}{\sum_{i=1}^{n_{EXT}}(y_i - \bar{y}_{TR})^2}$$

$$\mathrm{Q^2_{F2}} = 1 - \frac{\sum_{i=1}^{n_{EXT}}(y_i - \hat{y}_i)^2}{\sum_{i=1}^{n_{EXT}}(y_i - \bar{y}_{EXT})^2}$$

$$\mathrm{Q^2_{F3}} = 1 - \frac{\sum_{i=1}^{n_{EXT}}(y_i - \hat{y}_i)^2 / n_{TR}}{\sum_{i=1}^{n_{EXT}}(y_i - \bar{y}_{TR})^2 / n_{TR}}$$

Where, n_{TR} and n_{EXT} are the number of training and test set compounds and y_i and $\hat{y}_i$ are observed and predicted activity values and $\bar{y}_{EXT}$ is the mean of activity values of the external test set.

To verify if the predicted values and observed values matched, we calculated the Concordance Correlation Coefficient (CCC) as given below:

$$\mathrm{CCC} = \frac{2\sum_{i=1}^{n_{EXT}}(y_i - \bar{y})(\hat{y}_i - \hat{y}_{avg})}{\sum_{i=1}^{n_{EXT}}(y_i - \bar{y}_{TR})^2 + \sum_{i=1}^{n_{EXT}}(\hat{y}_i - \hat{y}_{avg})^2 + n_{EXT}(\hat{y}_i - \hat{y}_{avg})}$$

The mean absolute error (MAE) and r_m^2 by Roy et al. [3] are evaluated as:

$$\mathrm{MAE} = \frac{\sum_{i=1}^{n_{EXT}} |y_i - \hat{y}_{avg}|}{n_{EXT}}$$

$$r_m^2 = r^2(1 - \sqrt{r^2 - r_0^2})$$

Where, r^2 and r_0^2 are respectively the determination coefficients of the regression function, calculated using the experimental and the predicted data of the prediction set, forcing respectively the origin of the axis (r_0^2) or not (r^2).

Applicability domain:

The Applicability Domain is described in terms of the most relevant parameters to assess the applicability, reliability, and decidability of predictive models. In recent years, several AD methods have been adapted to assess 3D-QSAR models. One approach to check the AD of a model is by plotting the standard residuals against the leverage called the williams plot. The leverage values and standard residual values are evaluated using the Applicability domain toolbox in MATLAB (https://michem.unimib.it/download/matlab-toolboxes/applicability-domain-toolbox-for-matlab/) [4, 5].

The leverage values can be calculated as:

$$H = X(X^T X)^{-1} X^T$$

Where X is the model matrix while X^T is its transpose matrix.

The standard residuals can be calculated as shown below :

$$\delta = \frac{y_i - \hat{y}_i}{\sqrt{\sum_{i=1}^{n} \frac{(y_i - \hat{y}_i)^2}{(n - A - 1)}}}$$

Where y_i and $\hat{y}_i$ is the observed and predicted activity values of ith compound. The number of descriptors and the number of compounds are represented by *A* and *n* respectively.

Table S5. The SMILES structure of the 150 designed compounds.

**D01**	N([C@H]1C[C@H](N(CC1)Cc1ccc(Cl)cc1)C(C)C)c1c2cc[nH+]c2[nH]cc1C(=O)NC
**D02**	N([C@H]1C[C@H](N(CC1)Cc1ccc(Cl)cc1)C1CC1)c1c2cc[nH+]c2[nH]cc1C(=O)NC
**D03**	N([C@H]1C[C@H](N(CC1)Cc1ccc(Cl)cc1)C(CC)CC)c1c2cc[nH+]c2[nH]cc1C(=O)

	NC
**D05**	N([C@H]1C[C@H](N(CC1)Cc1ccc(Cl)cc1)C(C)(C)C)c1c2cc[nH+]c2[nH]cc1C(=O)NC
**D06**	N([C@H]1C[C@H](N(CC1)Cc1ccc(C(F)(F)F)cc1)C(C)(C)C)c1c2cc[nH+]c2[nH]cc1C(=O)NC
**D07**	N([C@H]1C[C@H](N(CC1)Cc1ccc(C)cc1)C(C)(C)C)c1c2cc[nH+]c2[nH]cc1C(=O)NC
**D08**	N([C@H]1C[C@H](N(CC1)Cc1ccc(C)cc1)C(C)C)c1c2cc[nH+]c2[nH]cc1C(=O)NC
**D09**	N([C@H]1C[C@H](N(CC1)Cc1ccc(C(C)(C)C)cc1)C(C)C)c1c2cc[nH+]c2[nH]cc1C(=O)NC
**D10**	N([C@H]1C[C@H](N(CC1)Cc1ccc(C2CC2)cc1)C(C)C)c1c2cc[nH+]c2[nH]cc1C(=O)NC
**D11**	N([C@H]1C[C@H](N(CC1)Cc1ccc(C2CCC2)cc1)C(C)C)c1c2cc[nH+]c2[nH]cc1C(=O)NC
**D12**	N([C@H]1C[C@H](N(CC1)Cc1ccc([C@H](C)CC)cc1)C(C)C)c1c2cc[nH+]c2[nH]cc1C(=O)NC
**D13**	N([C@H]1C[C@H](N(CC1)Cc1ccc(CC)c(c1)OC)C(C)C)c1c2cc[nH+]c2[nH]cc1C(=O)NC
**D14**	N([C@H]1C[C@H](N(CC1)Cc1ccc(CC)c(c1)CC)C(C)C)c1c2cc[nH+]c2[nH]cc1C(=O)NC
**D15**	N([C@H]1C[C@H](N(CC1)Cc1ccc(CCC)c(c1)CC)C(C)C)c1c2cc[nH+]c2[nH]cc1C(=O)NC
**D16**	N([C@H]1C[C@H](N(CC1)Cc1ccc(CCC)c(c1)C)C(C)C)c1c2cc[nH+]c2[nH]cc1C(=O)NC
**D17**	S(=O)(=O)([C@H]1C[C@H](N(CC1)Cc1ccc(CCC)c(c1)C)C(C)C)c1c2cc[nH+]c2[nH]cc1C(=O)NC
**D18**	S(=O)(=O)([C@H]1C[C@H](N(CC1)Cc1ccc(C)c(c1)C)C(C)C)c1c2cc[nH+]c2[nH]cc1C(=O)NC
**D19**	S(=O)(=O)([C@H]1C[C@H](N(CC1)Cc1ccc(C)cc1)C(C)C)c1c2cc[nH+]c2[nH]cc1C(=O)NC
**D20**	S(=O)(=O)([C@H]1C[C@H](N(CC1)Cc1ccc(Cl)cc1)C(C)C)c1c2cc[nH+]c2[nH]cc1C(=O)NC
**D21**	S(=O)(=O)([C@H]1C[C@H](N(CC1)Cc1ccc(Cl)cc1)C)c1c2cc[nH+]c2[nH]cc1C(=O)NC D21
**D22**	S(=O)(=O)([C@H]1C[C@H](N(CC1)Cc1ccc(C(F)(F)F)cc1)C)c1c2cc[nH+]c2[nH]cc1C(=O)NC
**D23**	S(=O)(=O)([C@H]1C[C@H](N(CC1)Cc1ccc(C(Cl)(Cl)Cl)cc1)C)c1c2cc[nH+]c2[nH]cc1C(=O)NC
**D24**	S(=O)(=O)([C@H]1C[C@H](N(CC1)Cc1ccc(N)cc1)C)c1c2cc[nH+]c2[nH]cc1C(=O)NC D24
**D25**	S(=O)(=O)([C@H]1C[C@H](N(CC1)Cc1ccc(F)cc1)C)c1c2cc[nH+]c2[nH]cc1C(=O)NC
**D26**	N([C@H]1[C@H]([C@H](N(CC1)Cc1ccc(Cl)cc1)C)C)c1c2cc[nH]c2ncc1C(=O)NC D201
**D27**	N([C@H]1[C@H](CN(CC1)Cc1ccc(Cl)cc1)C)c1c2cc[nH]c2ncc1C(=O)NC
**D28**	N([C@H]1[C@H](CN(CC1)Cc1ccc(Cl)cc1)[NH3+])c1c2cc[nH]c2ncc1C(=O)NC
**D29**	N([C@H]1[C@H](CN(CC1)Cc1ccc(Cl)cc1)[NH2+]C)c1c2cc[nH]c2ncc1C(=O)NC

**D30**	N([C@H]1[C@H](CN(CC1)Cc1ccc(Cl)cc1)[NH+](C)C)c1c2cc[nH]c2ncc1C(=O)NC
**D31**	N([C@H]1[C@H](CN(CC1)Cc1ccc(Cl)cc1)C(C)(C)C)c1c2cc[nH]c2ncc1C(=O)NC
**D32**	N([C@H]1[C@H]([C@H](N(CC1)Cc1ccc(Cl)cc1)C)C(C)(C)C)c1c2cc[nH]c2ncc1C(=O)NC
**D33**	N([C@H]1[C@H]([C@H](N(CC1)Cc1ccc(Cl)cc1)C)C(C)(C)C)c1c2cc[nH]c2ncc1C(=O)NC
**D34**	N([C@H]1[C@H]([C@H](N(CC1)Cc1ccc(Cl)cc1)C)C(C)C)c1c2cc[nH]c2ncc1C(=O)NC
**D35**	N([C@H]1[C@H]([C@H](N(CC1)Cc1ccc(Cl)cc1)C)N(C)C)c1c2cc[nH]c2ncc1C(=O)NC
**D36**	N([C@H]1[C@H]([C@H](N(CC1)Cc1ccc(Cl)cc1)N)N(C)C)c1c2cc[nH]c2ncc1C(=O)NC
**D37**	N([C@H]1[C@H]([C@H](N(CC1)Cc1ccc(Cl)cc1)N)C(C)C)c1c2cc[nH]c2ncc1C(=O)NC
**D38**	N([C@H]1[C@H]([C@H](N(CC1)Cc1ccc(Cl)cc1)N)C(C)(C)C)c1c2cc[nH]c2ncc1C(=O)NC
**D39**	N([C@H]1[C@H]([C@H](N(CC1)Cc1ccc(Cl)cc1)N)CC)c1c2cc[nH]c2ncc1C(=O)NC
**D40**	N([C@H]1[C@H]([C@H](N(CC1)Cc1ccc(Cl)cc1)C)C1CC1)c1c2cc[nH]c2ncc1C(=O)NC
**D41**	N([C@H]1[C@H]([C@H](N(CC1)Cc1ccc(Cl)cc1)N)C1CC1)c1c2cc[nH]c2ncc1C(=O)NC
**D42**	N([C@H]1[C@H]([C@H](N(CC1)Cc1ccc(Cl)cc1)CC)C1CC1)c1c2cc[nH]c2ncc1C(=O)NC
**D43**	N([C@H]1[C@H]([C@H](N(CC1)Cc1ccc(Cl)cc1)C(C)C)C1CC1)c1c2cc[nH]c2ncc1C(=O)NC
**D44**	N([C@H]1[C@H]([C@H](N(CC1)Cc1ccc(Cl)cc1)C(C)(C)C)C1CC1)c1c2cc[nH]c2ncc1C(=O)NC
**D45**	N([C@H]1[C@@H]([C@H](N(CC1)Cc1ccc(C)cc1)[C@@H](C)N)CC)c1c2cc[nH]c2ncc1C(=O)NC
**D46**	N([C@H]1[C@@H]([C@H](N(CC1)Cc1ccc(C)cc1)C(=C)N)C(C)C)c1c2cc[nH]c2ncc1C(=O)NC
**D47**	N([C@H]1[C@@H]([C@H](N(CC1)Cc1ccc(F)cc1)C(=C)N)C(C)C)c1c2cc[nH]c2ncc1C(=O)NC
**D47**	N([C@H]1[C@@H]([C@H](N(CC1)Cc1ccc(F)cc1)[C@@H](C)N)C(C)C)c1c2cc[nH]c2ncc1C(=O)NC
**D48**	N([C@H]1[C@@H]([C@H](N(CC1)Cc1ccc(F)cc1)[C@@H](C)N)N(C)C)c1c2cc[nH]c2ncc1C(=O)NC
**D49**	N([C@H]1[C@@H]([C@H](N(CC1)Cc1ccc(F)cc1)N(C)N)N(C)C)c1c2cc[nH]c2ncc1C(=O)NC
**D50**	N([C@H]1[C@@H]([C@H](N(CC1)Cc1ccc(F)cc1)N(C)N)C(C)C)c1c2cc[nH]c2ncc1C(=O)NC
**D51**	N([C@H]1C[C@H](N(CC1)Cc1cccc(c1C)C)C)c1c2cc[nH+]c2[nH]cc1C(=O)NC
**D52**	N([C@H]1C[C@H](N(CC1)Cc1ccccc1C)C)c1c2cc[nH+]c2[nH]cc1C(=O)NC
**D53**	N([C@H]1C[C@H](N(CC1)Cc1cccc(c1)C)C)c1c2cc[nH+]c2[nH]cc1C(=O)NC
**D53**	N([C@H]1C[C@H](N(CC1)Cc1cccc(c1)C)C(C)C)c1c2cc[nH+]c2[nH]cc1C(=O)NC
**D55**	N([C@H]1C[C@H](N(CC1)Cc1ccccc1C)C(C)C)c1c2cc[nH+]c2[nH]cc1C(=O)NC

**D56**	N([C@H]1C[C@H](N(CC1)Cc1cccc(c1)N)C(C)C)c1c2cc[nH+]c2[nH]cc1C(=O)NC
**D57**	N([C@H]1C[C@H](N(CC1)Cc1ccc(cc1)N)C(C)C)c1c2cc[nH+]c2[nH]cc1C(=O)NC
**D58**	N([C@H]1C[C@H](N(CC1)Cc1ccccc1N)C(C)C)c1c2cc[nH+]c2[nH]cc1C(=O)NC
**D59**	C(=O)([C@H]1C[C@H](N(CC1)Cc1ccc(cc1)Cl)C(C)C)c1c2cc[nH+]c2[nH]cc1C(=O)NC
**D60**	C(=O)([C@H]1C[C@H](N(CC1)Cc1ccc(cc1N)Cl)C(C)C)c1c2cc[nH+]c2[nH]cc1C(=O)NC
**D61**	C(=O)([C@H]1C[C@H](N(CC1)Cc1ccc(cc1N)C)C(C)C)c1c2cc[nH+]c2[nH]cc1C(=O)NC
**D62**	N([C@H]1C[C@H](N(CC1)Cc1cc(cc(c1)C)C)C(C)(C)C)c1c2cc[nH+]c2[nH]cc1C(=O)NC
**D63**	N([C@H]1C[C@H](N(CC1)Cc1c(ccc(c1)C)C)C(C)(C)C)c1c2cc[nH+]c2[nH]cc1C(=O)NC
**D64**	N([C@H]1C[C@H](N(CC1)Cc1c(ccc(c1)C)C)C(C)C)c1c2cc[nH+]c2[nH]cc1C(=O)NC
**D65**	N([C@H]1C[C@H](N(CC1)Cc1c(ccc(c1)O)C)C(C)C)c1c2cc[nH+]c2[nH]cc1C(=O)NC
**D66**	N([C@H]1C[C@H](N(CC1)Cc1c(ccc(c1)O)C(C)C)C(C)C)c1c2cc[nH+]c2[nH]cc1C(=O)NC
**D67**	N([C@H]1C[C@H](N(CC1)Cc1c(ccc(c1)N)C(C)C)C(C)C)c1c2cc[nH+]c2[nH]cc1C(=O)NC
**D68**	N([C@H]1C[C@H](N(CC1)Cc1cccc(c1)C(C)C)C(C)C)c1c2cc[nH+]c2[nH]cc1C(=O)NC
**D69**	N([C@H]1C[C@H](N(CC1)Cc1cccc(c1)C1CC1)C(C)C)c1c2cc[nH+]c2[nH]cc1C(=O)NC
**D70**	N([C@H]1C[C@H](N(CC1)Cc1cc(cc(c1)C)C)C(C)(C)C)c1c2cc[nH+]c2[nH]cc1C(=O)NC
**D71**	N([C@H]1C[C@H](N(CC1)Cc1c(ccc(c1)C)C)C(C)(C)C)c1c2cc[nH+]c2[nH]cc1C(=O)NC
**D72**	N([C@H]1C[C@H](N(CC1)Cc1ccc(cc1)F)C(C)C)c1c2cc[nH+]c2[nH]cc1C(=O)NC
**D73**	N([C@H]1C[C@H](N(CC1)Cc1cccc(c1)C(N)N)C(C)C)c1c2cc[nH+]c2[nH]cc1C(=O)NC
**D74**	N([C@H]1C[C@H](N(CC1)Cc1cccc2c1CCC2)C(C)C)c1c2cc[nH+]c2[nH]cc1C(=O)NC
**D75**	N([C@H]1C[C@H](N(CC1)Cc1cccc2c1CCC2)C(C)C)c1c2cc[nH+]c2[nH]cc1C(=O)NC
**D76**	N([C@H]1C[C@H](N(CC1)Cc1cccc2c1CCC2)C)c1c2cc[nH+]c2[nH]cc1C(=O)NC
**D77**	N([C@H]1[C@H]([C@H](N(CC1)Cc1ccc(Cl)cc1)[C@@H](C)N)C)c1c2cc[nH]c2ncc1C(=O)NC
**D78**	N([C@H]1[C@H]([C@H](N(CC1)Cc1ccc(Cl)cc1)[C@@H](C)N)CN)c1c2cc[nH]c2ncc1C(=O)NC
**D79**	N([C@H]1[C@H]([C@H](N(CC1)Cc1ccc(Cl)cc1)CN)CN)c1c2cc[nH]c2ncc1C(=O)NC
**D80**	N([C@H]1[C@H]([C@H](N(CC1)Cc1ccc(Cl)cc1)C(N)N)C)c1c2cc[nH]c2ncc1C(=O)NC
**D81**	N([C@H]1[C@H]([C@H](N(CC1)Cc1ccc(Cl)cc1)C(N)(N)N)CN)c1c2cc[nH]c2ncc1C(=O)NC
**D82**	N([C@H]1[C@H]([C@H](N(CC1)Cc1ccc(Cl)cc1)C(N)N)CN)c1c2cc[nH]c2ncc1C(

	=O)NC
**D83**	N([C@H]1[C@@H]2[C@H](N(CC1)Cc1ccc(Cl)cc1)[CH3]C[CH3]2)c1c2cc[nH]c2ncc1C(=O)NC
**D84**	N([C@H]1[C@@H](CN(C[C@@H]1C)Cc1ccc(Cl)cc1)C)c1c2cc[nH]c2ncc1C(=O)NC
**D85**	N([C@H]1[C@H]2[C@H](N(CC1)Cc1ccc(Cl)cc1)CCN2)c1c2cc[nH]c2ncc1C(=O)NC
**D86**	N([C@H]1[C@H]2[C@H](N(CC1)Cc1ccc(Cl)cc1)C[C@H](N2)C)c1c2cc[nH]c2ncc1C(=O)NC
**D87**	N([C@H]1[C@H]2[C@H](N(CC1)Cc1ccc(Cl)cc1)[NH2+][C@H](N2)C)c1c2cc[nH]c2ncc1C(=O)NC
**D88**	N([C@H]1[C@H]2[C@H](N(CC1)Cc1ccc(Cl)cc1)C[C@H](C2)C)c1c2cc[nH]c2ncc1C(=O)NC
**D89**	N([C@H]1[C@H]2[C@H](N(CC1)Cc1ccc(Cl)cc1)CC(C2)(C)C)c1c2cc[nH]c2ncc1C(=O)NC
**D90**	N([C@H]1[C@H]2[C@H](N(CC1)Cc1ccc(Cl)cc1)CC([NH2+]2)(C)C)c1c2cc[nH]c2ncc1C(=O)NC
**D91**	N([C@H]1[C@H]2[C@H](N(CC1)Cc1ccc(Cl)cc1)[NH2+]C([NH2+]2)(C)C)c1c2cc[nH]c2ncc1C(=O)NC
**D92**	N([C@H]1[C@H]2[C@H](N(CC1)Cc1ccc(Cl)cc1)[NH2+]C([NH2+]2)(N)N)c1c2cc[nH]c2ncc1C(=O)NC
**D93**	N([C@H]1[C@H]2[C@H](N(CC1)Cc1ccc(Cl)cc1)CC[C@@H]2C)c1c2cc[nH]c2ncc1C(=O)NC
**D94**	N([C@H]1[C@H]2[C@H](N(CC1)Cc1ccc(Cl)cc1)[C@@H](C[C@@H]2C)C)c1c2cc[nH]c2ncc1C(=O)NC
**D95**	N([C@H]1[C@H]2[C@H](N(CC1)Cc1ccc(Cl)cc1)C(CC2)(C)C)c1c2cc[nH]c2ncc1C(=O)NC
**D96**	N([C@H]1C[C@H](N(CC1)Cc1ccc(Cl)cc1)[C@@H](C(C)C)N)c1c2cc[nH]c2ncc1C(=O)NC
**D97**	N([C@H]1[C@@H]([C@H](N(CC1)Cc1ccc(Cl)cc1)[C@@H](C(C)C)N)C)c1c2cc[nH]c2ncc1C(=O)NC
**D98**	N([C@H]1[C@@H]([C@H](N(CC1)Cc1ccc(C)cc1)[C@@H](C(C)C)N)C)c1c2cc[nH]c2ncc1C(=O)NC
**D99**	N([C@H]1[C@@H]([C@H](N(CC1)Cc1ccc(C)cc1)[C@@H](C)N)C)c1c2cc[nH]c2ncc1C(=O)NC
**D100**	N([C@H]1[C@@H]([C@H](N(CC1)Cc1ccc(C)cc1)C(=C)N)C)c1c2cc[nH]c2ncc1C(=O)NC
**D101**	N([C@H]1C[C@H](N(CC1)Cc1cccc(c1C)C)C)c1c2cc[nH+]c2[nH]cc1C(=O)NC
**D102**	N([C@H]1C[C@H](N(CC1)Cc1cccc(c1N)N)C)c1c2cc[nH+]c2[nH]cc1C(=O)NC
**D103**	N([C@H]1C[C@H](N(CC1)Cc1cccc(c1)N)C)c1c2cc[nH+]c2[nH]cc1C(=O)NC
**D104**	N([C@H]1C[C@H](N(CC1)Cc1ccccc1N)C)c1c2cc[nH+]c2[nH]cc1C(=O)NC
**D105**	N([C@H]1C[C@H](N(CC1)Cc1ccccc1N(C)C)C)c1c2cc[nH+]c2[nH]cc1C(=O)NC
**D106**	N([C@H]1C[C@H](N(CC1)Cc1ccccc1NC)C)c1c2cc[nH+]c2[nH]cc1C(=O)NC
**D107**	N([C@H]1C[C@H](N(CC1)Cc1ccccc1CC)C)c1c2cc[nH+]c2[nH]cc1C(=O)NC
**D108**	N([C@H]1C[C@H](N(CC1)Cc1cccc(c1)N)C)c1c2cc[nH+]c2[nH]cc1C(=O)NC
**D109**	N([C@H]1C[C@H](N(CC1)Cc1cccc(c1)N(C)C)C)c1c2cc[nH+]c2[nH]cc1C(=O)NC
**D110**	N([C@H]1C[C@H](N(CC1)Cc1cccc(c1)NC)C)c1c2cc[nH+]c2[nH]cc1C(=O)NC
**D111**	N([C@H]1C[C@H](N(CC1)Cc1cccc(c1)NC)C(C)C)c1c2cc[nH+]c2[nH]cc1C(=O)N
**D112**	N([C@H]1C[C@H](N(CC1)Cc1cccc(c1)NC)C(C)(C)C)c1c2cc[nH+]c2[nH]cc1C(=

	O)NC
**D113**	N([C@H]1C[C@H](N(CC1)Cc1cccc(c1)N(C)C)C(C)(C)C)c1c2cc[nH+]c2[nH]cc1C(=O)NC
**D114**	N([C@H]1C[C@H](N(CC1)Cc1cccc(c1)N(C)C)C1CC1)c1c2cc[nH+]c2[nH]cc1C(=O)NC
**D115**	N([C@H]1C[C@H](N(CC1)Cc1cccc(c1)N(C)C)CN)c1c2cc[nH+]c2[nH]cc1C(=O)NC
**D116**	N([C@H]1C[C@H](N(CC1)Cc1cccc(c1)N(C)C)C(C)C)c1c2cc[nH+]c2[nH]cc1C(=O)N
**D117**	N([C@H]1C[C@H](N(CC1)Cc1cccc(c1)N(C)C)N)c1c2cc[nH+]c2[nH]cc1C(=O
**D118**	N([C@H]1C[C@H](N(CC1)Cc1cccc(c1)C(C)C)C(C)C)c1c2cc[nH+]c2[nH]cc1C(=O)N
**D119**	N([C@H]1C[C@H](N(CC1)Cc1cccc(c1)C(C)C)C(C)C)c1c2cc[nH+]c2[nH]cc1O
**D120**	N([C@H]1C[C@H](N(CC1)Cc1cccc(c1)C(C)C)C(C)C)c1c2cc[nH+]c2[nH]cc1N
**D121**	N([C@H]1C[C@H](N(CC1)Cc1ccc(cc1)Cl)C(C)C)c1c2cc[nH+]c2[nH]cc1N
**D122**	N([C@H]1C[C@H](N(CC1)Cc1ccc(cc1)Cl)C1CC1)c1c2cc[nH+]c2[nH]cc1N
**D123**	N([C@H]1C[C@H](N(CC1)Cc1ccc2c(c1)CCC2)C(C)C)c1c2cc[nH+]c2[nH]cc1C(=O)NC
**D125**	N([C@H]1C[C@H](N(CC1)Cc1ccc(cc1N)C)C(C)C)c1c2cc[nH+]c2[nH]cc1C(=O)NC
**D126**	N([C@H]1C[C@H](N(CC1)Cc1ccc(c(c1)C)C)C(C)C)c1c2cc[nH+]c2[nH]cc1C(=O)NC
**D127**	N([C@H]1C[C@H](N(CC1)Cc1ccc(c(c1)CC)CC)C(C)C)c1c2cc[nH+]c2[nH]cc1C(=O)NC
**D128**	N([C@H]1C[C@H](N(CC1)Cc1ccc(c(c1)N)C(C)C)C(C)C)c1c2cc[nH+]c2[nH]cc1C(=O)NC
**D129**	N([C@H]1C[C@H](N(CC1)Cc1ccc(c(c1)N)C(C)(C)C)C(C)C)c1c2cc[nH+]c2[nH]cc1C(=O)NC
**D131**	N([C@H]1C[C@H](N(CC1)Cc1ccc(C)c(c1)C(C)(C)C)C(C)(C)C)c1c2cc[nH+]c2[nH]cc1C(=O)N
**D133**	N([C@H]1C[C@H](N(CC1)Cc1ccc(C)c(c1)C(C)(C)C)C1CC1)c1c2cc[nH+]c2[nH]cc1C(=O)N
**D133**	N([C@H]1C[C@H](N(CC1)Cc1ccc(C)c(c1)C(C)(C)C)C1CC1)c1c2cc[nH+]c2[nH]cc1C(=O)N
**D135**	N([C@H]1C[C@H](N(CC1)Cc1ccc(C)c(c1)C(C)C)C(C)C)c1c2cc[nH+]c2[nH]cc1C(=O)N
**D136**	N([C@H]1C[C@H](N(CC1)Cc1cccc(c1)N1CC1)C(C)(C)C)c1c2cc[nH+]c2[nH]cc1C(=O)NC
**D137**	N([C@H]1C[C@H](N(CC1)Cc1cccc(c1)N1CC1)C(C)(C)C)c1c2cc[nH+]c2[nH]cc1C(=O)N
**D138**	N([C@H]1C[C@H](N(CC1)Cc1cccc(c1)N1CC1)C(C)C)c1c2cc[nH+]c2[nH]cc1C(=O)N
**D139**	N([C@H]1C[C@H](N(CC1)Cc1ccc(c(c1)C(C)C)C(C)C)C(C)C)c1c2cc[nH+]c2[nH]cc1C(=O)N
**D140**	N([C@H]1C[C@H](N(CC1)Cc1ccc(c(c1)C(C)C)C(C)C)C1CC1)c1c2cc[nH+]c2[nH]cc1C(=O)N
**D141**	N([C@H]1C[C@H](N(CC1)Cc1ccc(c(c1)N(C)C)N(C)C)C1CC1)c1c2cc[nH+]c2[nH]cc1C(=O)N
**D142**	N([C@H]1C[C@H](N(CC1)Cc1ccc(c(c1)N)N)C1CC1)c1c2cc[nH+]c2[nH]cc1C(=O)N

**D143**	N([C@H]1C[C@H](N(CC1)Cc1cccc(c1)NC)C1CC1)c1c2cc[nH+]c2[nH]cc1C(=O)N
**D144**	N([C@H]1C[C@H](N(CC1)Cc1cccc(c1)[C@H](C)CC)C1CC1)c1c2cc[nH+]c2[nH]cc1C(=O)N
**D145**	N([C@H]1C[C@H](N(CC1)Cc1ccc(C)c(c1)C(C)C)C1CCCC1)c1c2cc[nH+]c2[nH]cc1C(=O)N
**D146**	N([C@H]1C[C@H](N(CC1)Cc1ccc(C)c(c1)C(C)C)C1CCC1)c1c2cc[nH+]c2[nH]cc1C(=O)N
**D149**	N([C@H]1C[C@H](N(CC1)Cc1ccc(C(C)C)c(c1)C(C)(C)C)C(C)(C)C)c1c2cc[nH+]c2[nH]cc1C(=O)N
**D150**	N([C@H]1[C@@H]([C@H](N(CC1)Cc1ccc(F)cc1)[C@@H](C)N)C(C)C)c1c2cc[nH]c2ncc1C(=O)NC

References:

1. Gramatica P, Sangion A: **A historical excursus on the statistical validation parameters for QSAR models: a clarification concerning metrics and terminology**. *Journal of chemical information and modeling* 2016, **56**(6):1127-1131.
2. Shi LM, Fang H, Tong W, Wu J, Perkins R, Blair RM, Branham WS, Dial SL, Moland CL, Sheehan DM: **QSAR models using a large diverse set of estrogens**. *Journal of Chemical Information and Computer Sciences* 2001, **41**(1):186-195.
3. Roy K, Das RN, Ambure P, Aher RB: **Be aware of error measures. Further studies on validation of predictive QSAR models**. *Chemometrics and Intelligent Laboratory Systems* 2016, **152**:18-33.
4. Sahigara F, Mansouri K, Ballabio D, Mauri A, Consonni V, Todeschini R: **Comparison of different approaches to define the applicability domain of QSAR models**. *Molecules* 2012, **17**(5):4791-4810.
5. Sahigara F, Ballabio D, Todeschini R, Consonni V: **Assessing the validity of QSARs for ready biodegradability of chemicals: an applicability domain perspective**. *Current computer-aided drug design* 2014, **10**(2):137-147.
